# Tubular CPT1A deletion minimally affects aging and chronic kidney injury

**DOI:** 10.1172/jci.insight.171961

**Published:** 2024-03-22

**Authors:** Safaa Hammoud, Alla Ivanova, Yosuke Osaki, Steven Funk, Haichun Yang, Olga Viquez, Rachel Delgado, Dongliang Lu, Melanie Phillips Mignemi, Jane Tonello, Selene Colon, Louise Lantier, David H. Wasserman, Benjamin D. Humphreys, Jeffrey Koenitzer, Justin Kern, Mark de Caestecker, Toren Finkel, Agnes Fogo, Nidia Messias, Irfan J. Lodhi, Leslie S. Gewin

**Affiliations:** 1 Division of Nephrology and Hypertension, Department of Medicine, Washington University in St. Louis, St. Louis, Missouri, USA.; 2 Division of Nephrology and Hypertension, Department of Medicine, and; 3 Department of Pathology, Microbiology, and Immunology, Vanderbilt University Medical Center, Nashville, Tennessee, USA.; 4 Division of Endocrinology, Department of Medicine, Washington University in St. Louis, St. Louis, Missouri, USA.; 5 Molecular Physiology and Biophysics, Vanderbilt University, Nashville, Tennessee, USA.; 6 Division of Pulmonary Critical Care Medicine, Department of Medicine, Washington University in St. Louis, St. Louis, Missouri, USA.; 7 Aging Institute, Department of Medicine, University of Pittsburgh School of Medicine, Pittsburgh, Pennsylvania, USA.; 8 Department of Pathology and Immunology, Washington University in St. Louis, St. Louis, Missouri, USA.; 9 Department of Medicine, Veterans Affairs Hospital, St. Louis, Missouri, USA.

**Keywords:** Metabolism, Nephrology, Chronic kidney disease, Fatty acid oxidation

## Abstract

Kidney tubules use fatty acid oxidation (FAO) to support their high energetic requirements. Carnitine palmitoyltransferase 1A (CPT1A) is the rate-limiting enzyme for FAO, and it is necessary to transport long-chain fatty acids into mitochondria. To define the role of tubular CPT1A in aging and injury, we generated mice with tubule-specific deletion of *Cpt1a* (*Cpt1a*^CKO^ mice), and the mice were either aged for 2 years or injured by aristolochic acid or unilateral ureteral obstruction. Surprisingly, *Cpt1a*^CKO^ mice had no significant differences in kidney function or fibrosis compared with wild-type mice after aging or chronic injury. Primary tubule cells from aged *Cpt1a*^CKO^ mice had a modest decrease in palmitate oxidation but retained the ability to metabolize long-chain fatty acids. Very-long-chain fatty acids, exclusively oxidized by peroxisomes, were reduced in kidneys lacking tubular CPT1A, consistent with increased peroxisomal activity. Single-nuclear RNA-Seq showed significantly increased expression of peroxisomal FAO enzymes in proximal tubules of mice lacking tubular CPT1A. These data suggest that peroxisomal FAO may compensate in the absence of CPT1A, and future genetic studies are needed to confirm the role of peroxisomal β-oxidation when mitochondrial FAO is impaired.

## Abstract 2

CPT1A is the rate-limiting enzyme for fatty acid metabolism, but deleting CPT1A in tubules has minimal effect on aging and chronic kidney disease.

## Graphical abstract

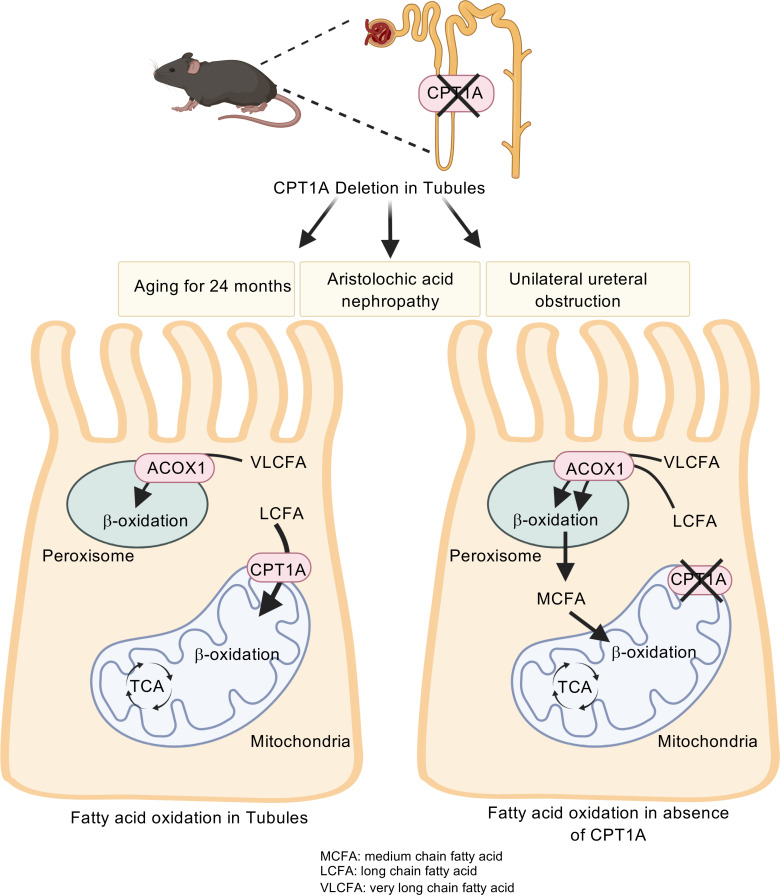

## Introduction

The kidney is a highly metabolically active organ, primarily due to the energy demands of the proximal tubules. The large water and solute reabsorptive capacity of proximal tubules necessitates high rates of ATP production. Thus, proximal tubules have large amounts of mitochondria to generate ATP and preferentially use fatty acid oxidation (FAO), which produces more ATP than glucose, as their preferred energy substrate. Impaired FAO has been described in both acute and chronic kidney injury (1, 2). Reduced FAO not only reflects kidney injury, but also is thought to play an important role in the pathogenesis of kidney injury through diminished ATP, accumulation of intracellular lipids, and reduced mitochondrial function. These effects all promote tubular atrophy and tubulointerstitial fibrosis, the hallmarks of chronic kidney disease (CKD) progression (1, 3, 4).

The aging kidney shares many features of CKD, including glomerulosclerosis, tubulointerstitial fibrosis, and tubular atrophy (5). The pathophysiology of age-associated renal fibrosis is multifactorial and includes the progressive loss of nephrons, cell senescence, inflammation, and dysfunctional mitochondria with ROS production (6–8). Altered metabolism may also play a role in aging-mediated pathological changes in the kidney, as proteins involved in FAO were decreased in the aged rat kidney (9, 10). PPARα transcriptionally upregulates many genes involved in FAO, and aged *Ppara^–/–^* mice had worsened kidney fibrosis (9), suggesting that reduced FAO may be linked mechanistically to aged-induced renal fibrosis.

Carnitine palmitoyltransferase 1 (CPT1) is the rate-limiting enzyme for mitochondrial FAO. CPT1 is part of the carnitine shuttle and is required for long-chain fatty acids (LCFA) to enter the mitochondria for β-oxidation and subsequent entry into the tricarboxylic acid cycle. There are 3 CPT1 isoforms (A, B, C), but CPT1A is the primary isoform expressed in kidney tubules (11, 12). CPT1A expression is reduced in both aged and injured kidneys (1, 9), but whether this decreased expression plays a role in the pathophysiology of the aging or injured kidney is unclear. To address this question, we selectively deleted *Cpt1a* in renal tubules using the inducible Pax8-rTTA;tetO-Cre mice (13) and either aged the mice for 2 years or subjected them to the unilateral ureteral obstruction (UUO) or aristolochic acid nephropathy (AAN) chronic kidney injury models.

## Results

### Tubular CPT1A plays a minor role in function and fibrosis of the aged kidney.

**Figure 1:**
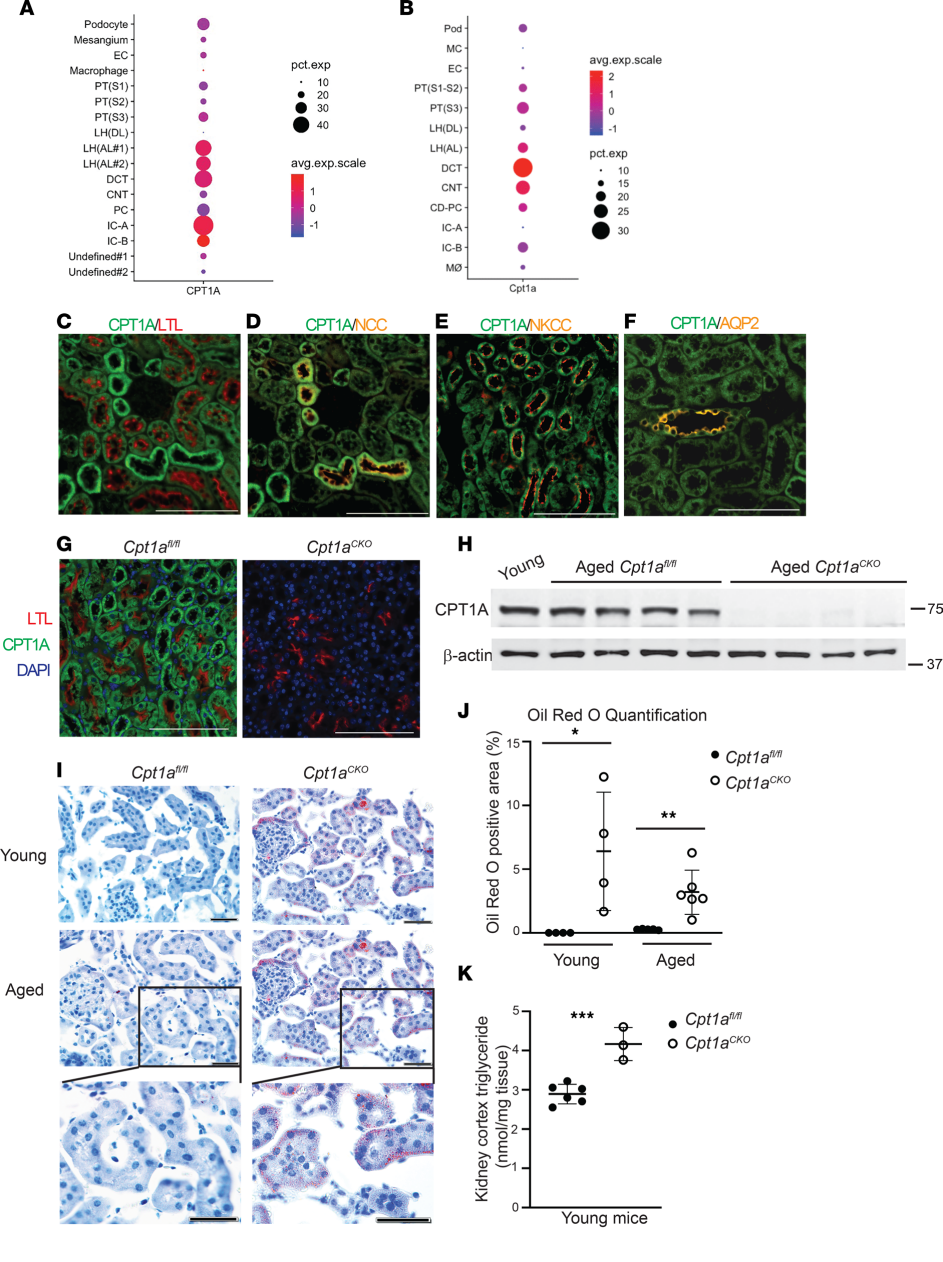
*Cpt1a^CKO^* mice have robust recombination and increased lipid accumulation. Single-cell transcriptomics data from Kidney Interactive Transcriptomics (https://humphreyslab.com/SingleCell/) shows gene expression of *CPT1A* (**A**) and *Cpt1a* (**B**) in normal human and murine kidneys, respectively. (**C**) CPT1A protein expression in young murine kidneys colocalizes with proximal tubules (LTL). Strongly CPT1A^+^ and LTL^–^ tubules stained for Na/Cl cotransporter (NCC), consistent with high CPT1A expression in the distal convoluted tubule (**D**). There was also colocalization of CPT1A with the thick ascending limb as marked by Na/K/Cl cotransporter (NKCC) and collecting ducts as shown by aquaporin 2 (AQP2) (**E** and **F**). CPT1A protein expression was efficiently blocked in *Cpt1a^CKO^* mice shown by IF in young mice (**G**) and cortical tissue immunoblots from aged mice with β-actin as loading control (*n* = 4) (**H**). Representative frozen sections, with Oil Red O staining lipid droplets in red, with quantification of percentage of positive area (*n* = 4–6) (**I** and **J**). Triglyceride content within kidneys, with each dot representing a different kidney (*n* = 3–6) (**K**). Results are shown as the mean ± SD. *P < 0.05, **P < 0.01, ***P < 0.001. Unpaired *t* test between the 2 genotypes was used to detect statistical significance. Scale bar: 100 μM (**C**–**G**); 50 μM (**I**).

FAO is the primary energy substrate for proximal tubules, but CPT1A is ubiquitously expressed throughout the kidney tubules (12, 14). Single-cell transcriptomics data from Kidney Interactive Transcriptomics (https://humphreyslab.com/SingleCell/) and ATLAS-D2K (https://www.rebuildingakidney.org/id/14-4KPM) unexpectedly showed that the highest gene expression was located in distal tubules in both murine and human kidneys (Figure 1, A and B) (12, 15, 16). We confirmed CPT1A protein expression in the proximal tubule and other nephron segments (Figure 1, C–F). Given CPTA1’s extensive tubular expression, we generated mice with inducible, tubule deletion of CPT1A (*Cpt1a*^CKO^ mice). Robust recombination was confirmed in young and 2-year-old mice (Figure 1, G and H). Young *Cpt1a*^CKO^ mice had about 20% larger kidneys without significant changes in body weight, and this difference in kidney sizes persisted in aged mice (Supplemental Figure 1; supplemental material available online with this article; https://doi.org/10.1172/jci.insight.171961DS1). CPT1A is required for LCFA entry into mitochondria and subsequent oxidation, so we postulated that its deletion may lead to accumulation of fatty acids within the tubules. Oil Red O staining on frozen sections demonstrated that both young and aged *Cpt1a*^CKO^ mice had significantly increased tubular lipid droplet accumulation compared with floxed controls, a finding confirmed by increased triglyceride content within *Cpt1a*^CKO^ kidney tissue (Figure 1, I–K).

**Figure 2:**
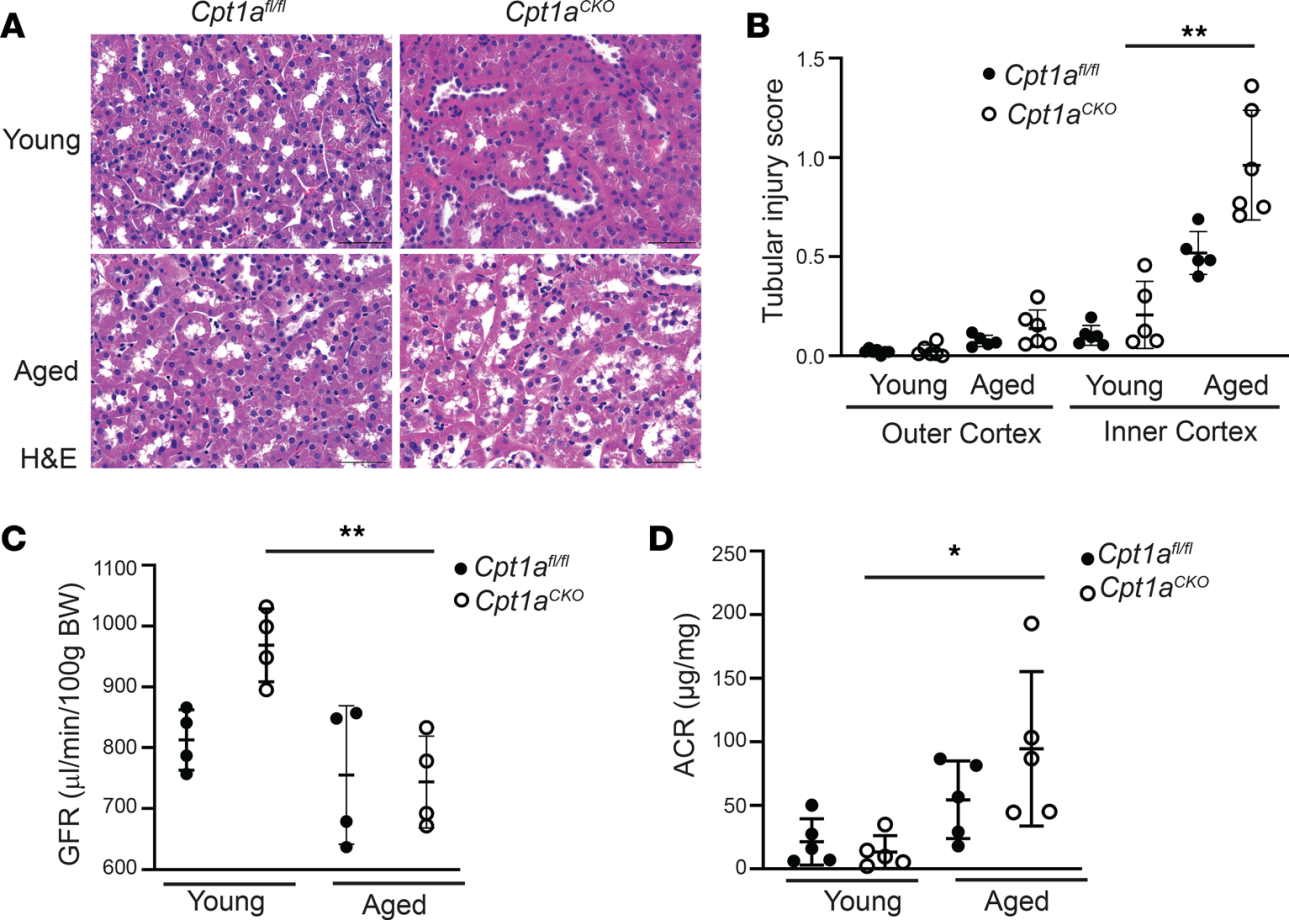
Deletion of tubular *Cpt1a* does not markedly impair kidney function in aging. Representative H&E staining of young and aged *Cpt1a^CKO^* and floxed mice with quantification of tubular injury (see Methods) in the outer and inner cortex (*n* = 5–6) (**A** and **B**). Transdermal glomerular filtration rate (tGFR) measured by FITC-sinistrin (*n* = 4) (**C**) and urinary albumin-to-creatinine ratio (ACR) (*n* = 5) (**D**) of young and old mice by genotype. Results are shown as the mean ± SD. *P < 0.05, **P < 0.01. Unpaired *t* test between the 2 genotypes was used to detect statistical significance between the 2 genotypes. For multiple comparisons in **C** and **D**, 1-way ANOVA was performed followed by Šidák’s multiple comparisons test. Scale bar: 50 μM.

**Figure 3:**
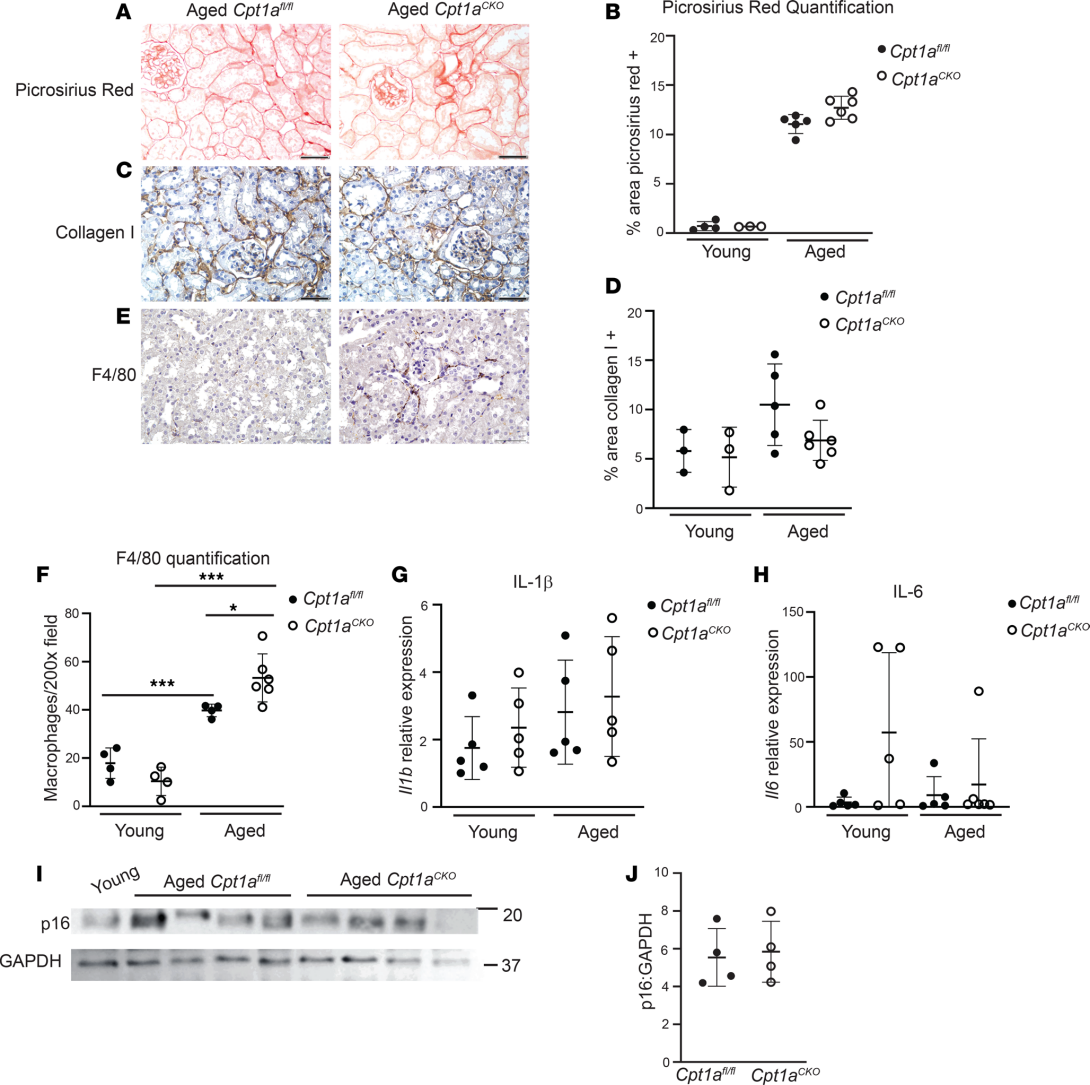
Aged *Cpt1a^CKO^* mice have increased inflammation but not increased fibrosis. No differences in fibrosis between aged *Cpt1a^CKO^* mice and floxed controls were observed by Picrosirius red staining (**A**) and quantification (*n* = 3–5) (**B**) or collagen I staining (**C**) and quantification (*n* = 3–6) (**D**). Aged *Cpt1a^CKO^* mice had increased macrophages, measured by immunohistochemistry for F4/80, shown in brown and quantified (*n* = 4–6) (**E** and **F**). Gene expression of IL-1β (*Il1b*, *n* = 5) and IL-6 (*Il6*, *n* = 5) in renal cortices are quantified by qPCR and normalized to *Gapdh* (**G** and **H**). Renal cortices were immunoblotted for p16 and GAPDH (loading control) and quantified (*n* = 4) using ImageJ (**I** and **J**). The use of GAPDH as loading control was validated against β-actin in Supplemental Figure 4. Data are shown as the mean ± SD. *P < 0.05, ***P < 0.001. One-way ANOVA was performed followed by Šidák’s multiple comparisons test for **F**, and unpaired *t* test between the 2 genotypes was used to measure significance in **B**, **G**, **H**, and **J**. Scale bar: 50 μM.

The aged *Cpt1a*^CKO^ mice had no differences in tubule injury in the outer cortex but had increased tubule injury scores (see Methods) in the inner cortex (Figure 2, A and B). No significant glomerular changes were noted between the aged genotypes. The *Cpt1a*^CKO^ mice had a more significant drop in transdermal glomerular filtration rate (tGFR) and increase in albuminuria with aging compared with the floxed controls, but there were no significant differences in tGFR or albuminuria between the genotypes in aged mice (Figure 2, C and D). Picrosirius red staining (Figure 3, A and B, and Supplemental Figure 2) showed a significant (WT old versus WT young, *P* < 0.0001; KO old versus KO young, *P* < 0.0001) increase in fibrosis in aged mice compared with young mice, but no significant differences in staining between the aged *Cpt1a*^CKO^ mice and floxed controls. Consistent with this, collagen I staining on aged kidney tissue showed no differences between *Cpt1a*^CKO^ and wild type mice (Figure 3, C and D, and Supplemental Figure 2). These data suggest that deleting tubular Cpt1a causes modest tubular injury but does not markedly affect function or fibrosis in the aged kidney.

### Tubular CPT1A deletion increases inflammation but does not affect senescence.

*Cpt1a*^CKO^ mice had increased lipid accumulation (Figure 1, I–K), which is associated with inflammation, and macrophage infiltration (F4/80^+^ cells) was increased in aged *Cpt1a*^CKO^ mice versus *Cpt1a*^fl/fl^ mice (Figure 3, E and F). Another potential cause of inflammation in aged mice is senescence, a state of irreversible cell cycle arrest associated with inflammation. We measured *Il1b* and *Il6* gene expression as well as p16 protein expression, all commonly used markers of senescence, but found no differences between aged *Cpt1a*^CKO^ and *Cpt1a*^fl/fl^ mice (Figure 3, G–J). Thus, aged *Cpt1a*^CKO^ mice have increased fatty acid accumulation and macrophage infiltration but not senescence compared with aged controls.

### Cpt1a^CKO^ mice do not have increased injury or fibrosis after CKD models.

**Figure 4:**
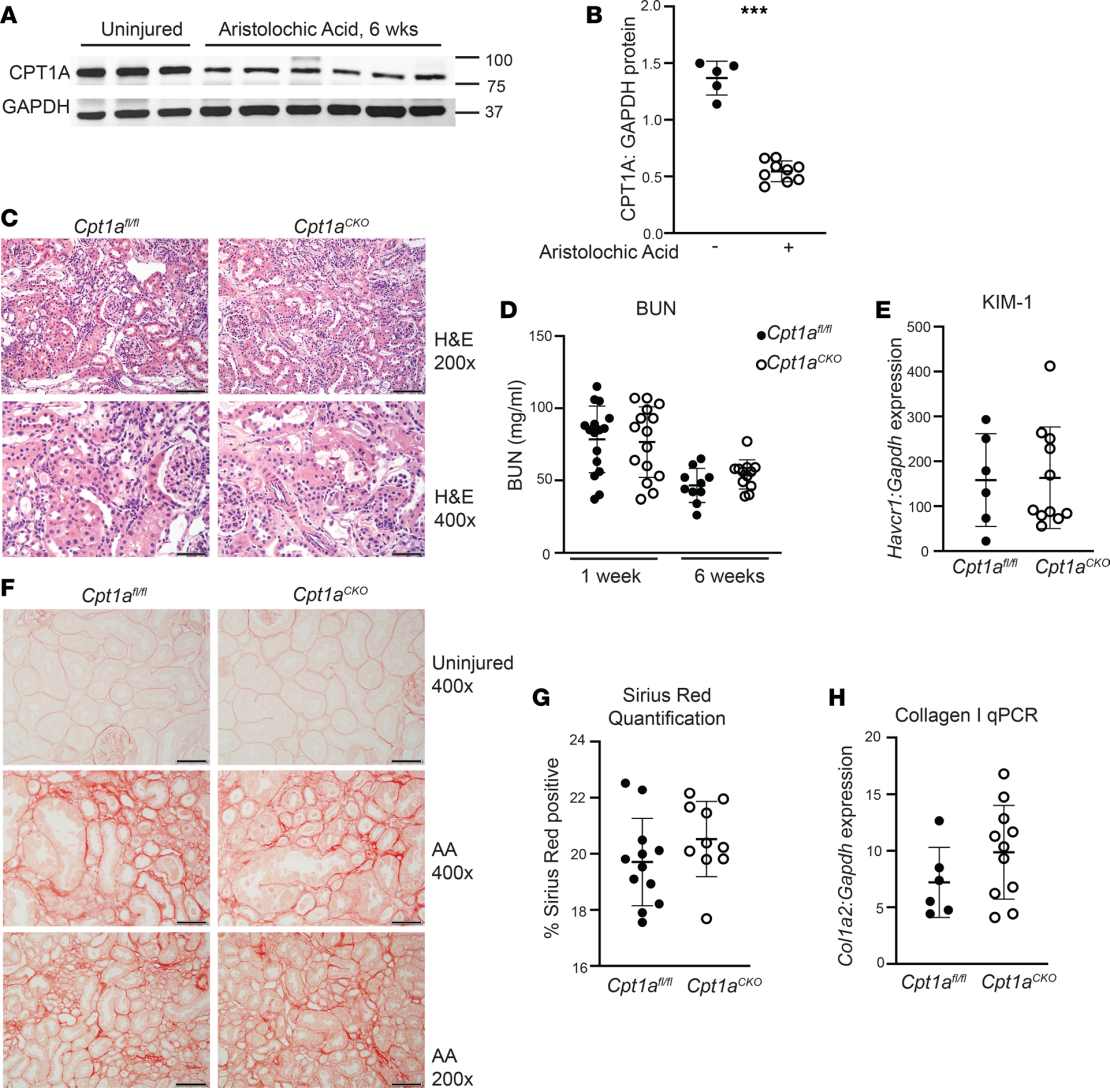
Tubular injury and fibrosis are not increased in *Cpt1a^CKO^* mice after aristolochic acid nephropathy. Immunoblots of renal cortices from uninjured and injured (aristolochic acid nephropathy [AAN]) mice detecting CPT1A expression (values are shown in kDa) (**A**), which was quantified with GAPDH as loading control (*n* = 5–9) (**B**). (**C**) H&E images of AAN-injured mice, with blood urea nitrogen (BUN) at both 1 and 6 weeks (*n* = 10–15) after aristolochic acid injections (**D**). Gene expression for KIM-1 (*Havcr1*, *n* = 6–10) measured in kidney tissue with qPCR and normalized to *Gapdh* (**E**). Picrosirius red staining and quantification (*n* = 10–12) (**F** and **G**) in injured kidneys and Col1a2 (*collagen I*, *n* = 6–12) gene expression by qPCR in AAN-injured cortices (**H**). Data are shown as the mean ± SD.***P < 0.001. Unpaired *t* test between the 2 genotypes was used to detect statistical significance. Scale bar: 50 μM (**C**, bottom, and **F**, top and middle); 100 μM (**C**, top, and **F**, bottom). Original magnification, ×200 (**C**, top, and **F**, bottom); ×400 (**C**, bottom, and **F**, top and middle).

Kidney overexpression of CPT1A was protective in several CKD models (3), so we assessed whether its deletion would be detrimental in chronic injury. AAN targets the proximal tubules and leads to CKD in both humans and rodents (17, 18). CPT1A expression was significantly suppressed 6 weeks after aristolochic acid administration (Figure 4, A and B). Both *Cpt1a*^CKO^ and *Cpt1a*^fl/fl^ mice had increased tubular injury and inflammation 6 weeks after AAN, with elevated blood urea nitrogen levels at 1 and 6 weeks after injury, but there was no difference in renal function between genotypes (Figure 4, C and D). In addition, there were no genotype differences in kidney injury molecule (KIM-1), a marker of proximal tubular injury, in kidney tissue (Figure 4E). Sirius red staining was increased in AAN-injured tissue, but there were no differences in Sirius red staining or collagen I gene expression between genotypes (Figure 4, F–H). Thus, tubular deletion of *Cpt1a* did not worsen tubule injury or fibrosis in the AAN model of CKD.

**Figure 5:**
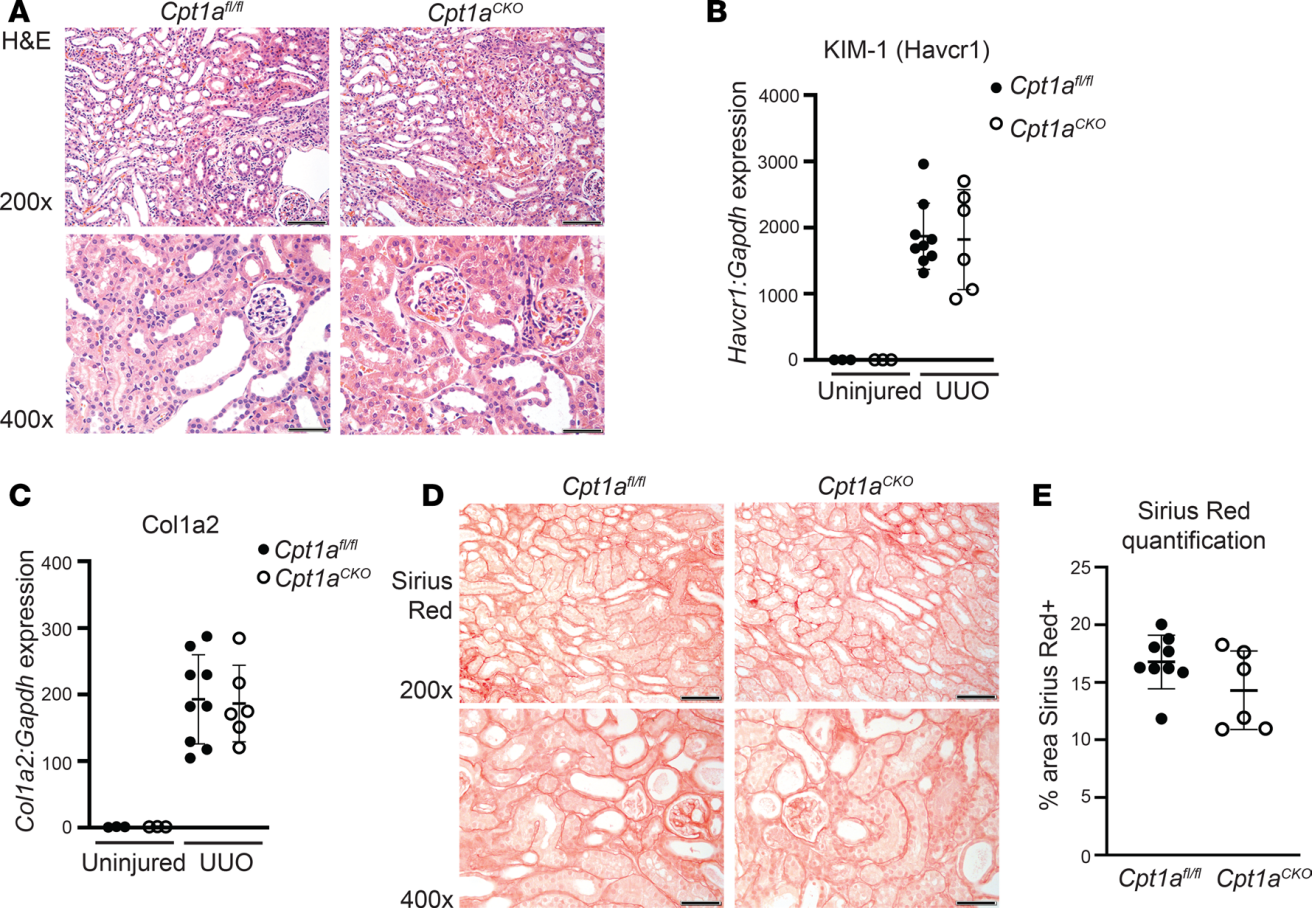
Deleting tubular *Cpt1a* does not alter fibrosis after UUO. H&E following 7 days of unilateral ureteral obstruction (UUO) (**A**). KIM-1 (*Havcr1*, *n* = 6–9) gene expression by qPCR of injured and uninjured tissue (**B**). Collagen I (*Col1a2*) gene expression (**C**) and Picrosirius red staining with quantification (*n* = 6–9) (**D** and **E**) after UUO injury. Data are shown as the mean ± SD. Scale bar: 50 μM (bottom rows); 100 μM (top rows). Original magnification: ×400 (bottom rows); ×200 (top rows).

*Cpt1a*^CKO^ and *Cpt1a*^fl/fl^ mice were also injured by UUO, the classic model of tubulointerstitial fibrosis in which CPT1A decreases after injury (19). At 7 days after UUO, the mice were sacrificed and both genotypes had increased tubule dilation and inflammation, but no differences in *KIM-1* were seen by qPCR between genotypes (Figure 5, A and B). Similarly, collagen I gene expression and Sirius red expression were increased in the UUO-injured kidneys, but there was no difference between genotypes (Figure 5, C–E). In summary, deletion of *Cpt1a* in renal tubules did not worsen response to either AAN- or UUO-induced CKD.

### Aged Cpt1a^–/–^ tubule cells have altered metabolism but are able to oxidize LCFA.

**Figure 6:**
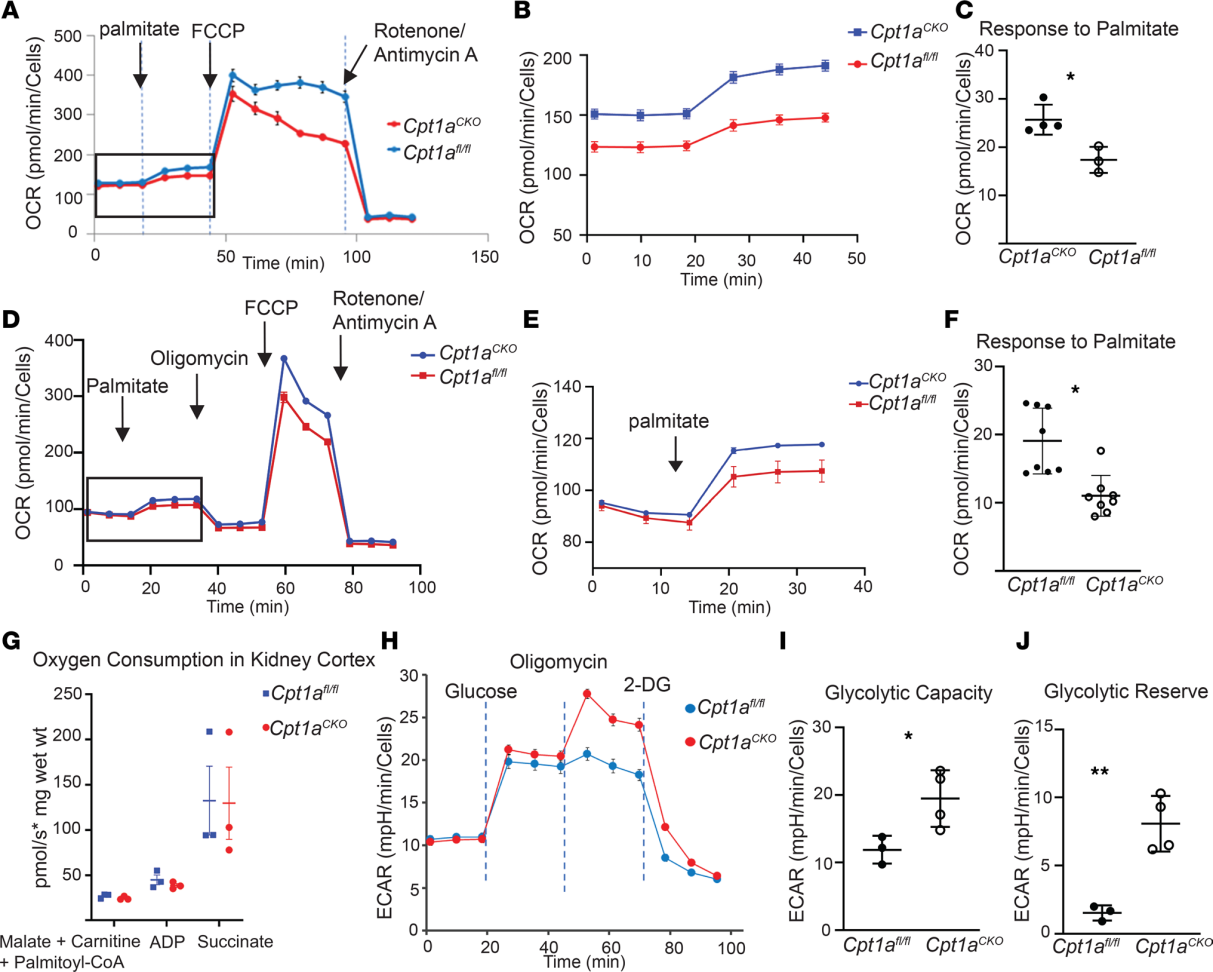
Primary proximal tubule–enriched cells lacking *Cpt1a* have altered metabolism but are still able to metabolize LCFA. Representative data from Seahorse analysis show oxygen consumption rate (OCR) after treatment with palmitate, FCCP, and antimycin A/rotenone in primary proximal tubule–enriched cells (PT cells) from aged mice (**A**). An expanded view (boxed area in **A**) of the OCR response to palmitate is shown (**B**). Average OCR responses to palmitate from *Cpt1a^fl/fl^* (*n* = 4) and *Cpt1a^CKO^* (*n* = 3) mice are quantified (**C**). Representative tracing from PT cells isolated from young mice showing OCR after palmitate, oligomycin, FCCP, and antimycin A/rotenone (**D**) with an expanded view of the response to palmitate (**E**) and quantification of this palmitate response with *n* = 8 per genotype (**F**). Kidney tissues from young *Cpt1a^fl/fl^* and *Cpt1a^CKO^* mice were used to measure oxygen consumption in response to palmitoyl-CoA, a LCFA (**G**). Each dot represents a separate kidney (*n* = 3), and oxygen consumption is measured as picomole per second per mg of kidney (wet weight). Representative data from a glycolysis test measuring extracellular acidification rate (ECAR) are shown (**H**). Oligomycin is used to detect maximal glycolytic capacity, and 2-DG blocks glycolytic acidification to define the glucose-dependent ECAR (**I**). Glycolytic capacity of primary PT cells from young and old mice (each dot represents a separate mouse *n* = 3–4) and glycolytic reserve (the ECAR value after oligomycin minus that after the addition of glucose) are shown (**J**). Results are shown as the mean ± SD. *P < 0.05, **P < 0.01. Unpaired *t* test between the 2 genotypes was used to detect statistical significance. FCCP, carbonyl cyanide-4 (trifluoromethoxy) phenylhydrazone; 2-DG, 2-deoxy-glucose; LCFA, long-chain fatty acid.

CPT1A, a critical component of the carnitine shuttle that is necessary for movement of LCFA into mitochondria, is considered the rate-limiting enzyme for FAO (11). The accumulation of lipid droplets within tubules of *Cpt1a*^CKO^ mice also suggested a defect in fatty acid metabolism, so we interrogated metabolic changes in primary tubular cells generated from aged *Cpt1a*^CKO^ and *Cpt1a*^fl/fl^ mice. We generated primary proximal tubule–enriched cell populations (primary PT cells) as previously described (20, 21). Primary PT cells were grown in low glucose-containing media and used for bioflux analysis within 5 days of plating to limit glycolytic conversion. Primary PT cells from aged *Cpt1a*^CKO^ mice had a 30% reduced response to palmitate, a LCFA, measured by oxygen consumption rate (OCR) (Figure 6, A–C). Etomoxir, a chemical CPT1 inhibitor commonly used in Seahorse, has been found to have off-target effects at doses above 5 μM, leading to reduced OCR in a FAO-independent manner (22–24). Thus, many effects of etomoxir are not dependent upon CPT1 or FAO.

Oxidative metabolism analyses performed on primary PT cells from young mice also showed a consistent decrease in palmitate response (Figure 6, D–F). Though LCFA-dependent OCR was significantly impaired in primary PT cells from *Cpt1a^CKO^* mice, these cells retained the ability to metabolize LCFA. We further investigated using high-resolution respirometry with Oroboros Oxygraph and showed that kidney cortical tissue ex vivo from young *Cpt1a^CKO^* mice and floxed controls had no difference in LCFA oxidation (Figure 6G). Glycolysis was also analyzed, and primary PT cells from aged *Cpt1a^CKO^* mice demonstrated increased glycolytic capacity and glycolytic reserve, reflecting the cells’ ability to augment glycolysis in response to an energetic demand, compared with that of aged floxed controls (Figure 6, H–J). Thus, tubular cells from aged *Cpt1a^CKO^* mice had a modest but significant impairment in LCFA metabolism and concomitant increased glycolytic capacity. The fact that CPT1A deletion impairs, but not prevents, LCFA suggests that other compensatory mechanisms may be present. However, CPT1A is not required for LCFA oxidation in either primary cells or tissue ex vivo.

**Figure 7:**
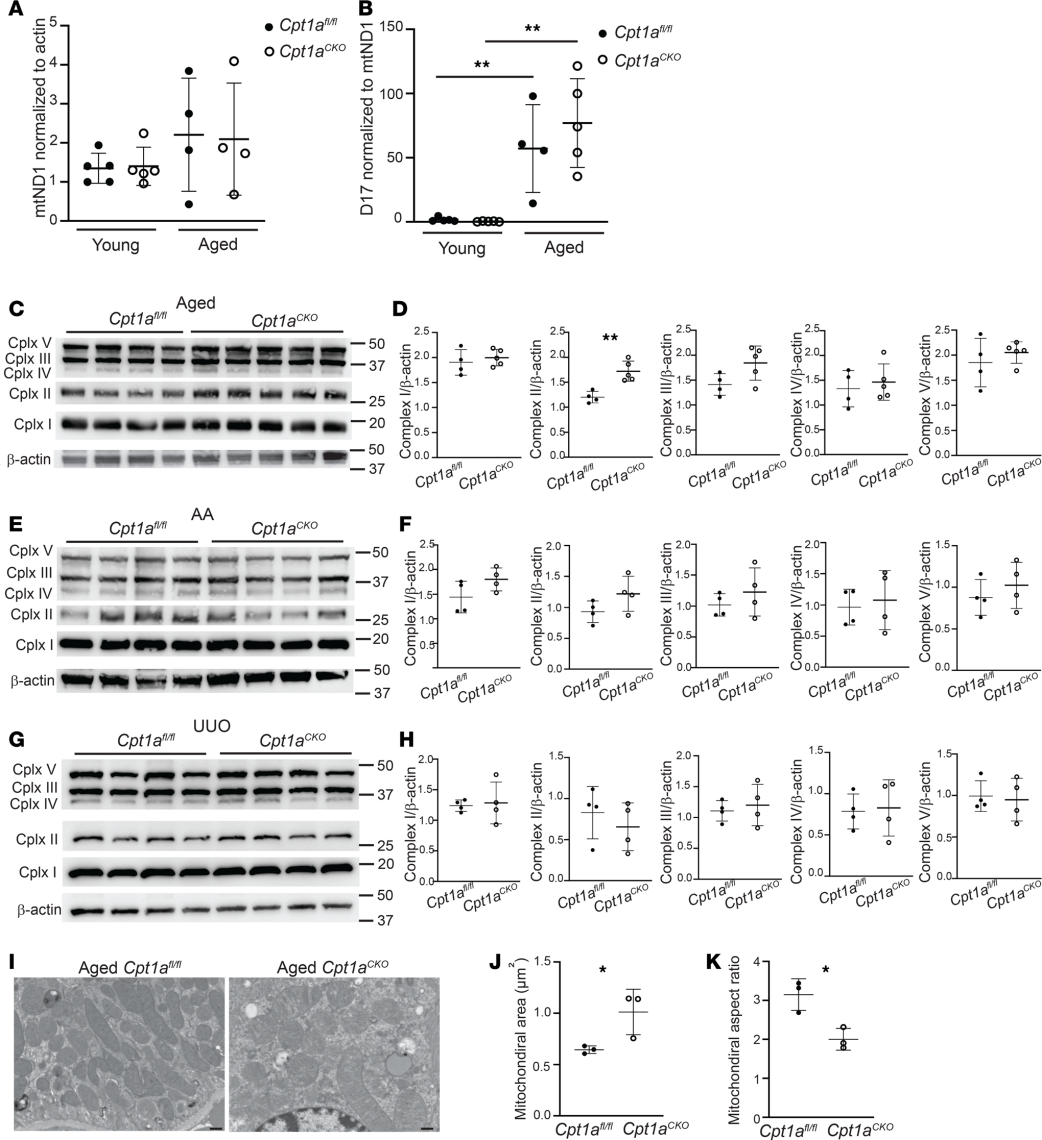
Assessment of *Cpt1a* tubular deletion on mitochondria. Mitochondrial DNA copy number was measured using mitochondrial-encoded ND1 gene expression and normalized to nuclear-encoded actin (*n* = 4–5) (**A**). The common mitochondrial mutation, *D17*, was measured using qPCR and normalized to mitochondrial *ND1* expression (*n* = 4–5) (**B**). Mitochondrial complexes were measured and quantified using immunoblots on cortical kidney tissue using ImageJ from aged (**C** and **D**), aristolochic acid–treated (AA-treated) (**E** and **F**), and UUO-injured mice (*n* = 4–5) (**G** and **H**). Representative electron micrograph images are shown (**I**) from aged mice, with quantification of area and aspect ratio (**J** and **K**) (see Methods). Each dot represents an average value for 1 mouse (*n* = 3). Results are shown as the mean ± SD. *P < 0.05; **P < 0.01. Unpaired *t* test between the 2 genotypes was used to detect statistical significance, except for **A** and **B**, where 1-way ANOVA was performed, followed by Šidák’s multiple comparisons. Scale bar: 500 nM.

Metabolism and mitochondrial function are closely intertwined. Mitochondrial DNA copy number, an approximation of mitochondrial biogenesis and function, was also unchanged in the aged mice between genotypes (Figure 7A). D17 is considered the “common mitochondrial mutation” and was significantly increased in aged compared with young mice (Figure 7B) (25). However, there were no changes in mitochondrial DNA mutation number between genotypes. Expression of mitochondrial complexes, a surrogate for mitochondrial mass, was not decreased in the kidneys from *Cpt1a^CKO^* mice compared with floxed controls (Figure 7, C–H). There was a slight but significant increase in complex II in the aged *Cpt1a^CKO^* kidneys but otherwise no changes after AAN or UUO. Electron microscopy (EM) images showed a significant increase in mitochondrial area in the *Cpt1a^CKO^* mice as well as decreased mitochondrial aspect ratio (AR), suggesting a more rounded mitochondrial morphology that could be consistent with fragmentation in the *Cpt1a^CKO^* mice (Figure 7, I–K). As dysfunctional mitochondrial can be important sources of oxidative stress, we assessed this by immunostaining for 4-hydroxynonenal, a product of lipid peroxidation. Although staining in aged mice was greater than that in young mice, there were no differences between the genotypes (Supplemental Figure 3). Urine F2 isoprostanes, another indicator of oxidative stress, were not significantly changed in aged *Cpt1a^CKO^* mice compared with *Cpt1a^fl/fl^* mice (Supplemental Figure 3). Taken together, there were slight structural changes in mitochondria from Cpt1a^fl/fl^ mice, but no differences in mitochondrial mass, mutations, or oxidative stress in aged mice lacking tubular CPT1A compared with littermate controls.

### PPARα and peroxisomes may compensate in the absence of CPT1A.

**Figure 8:**
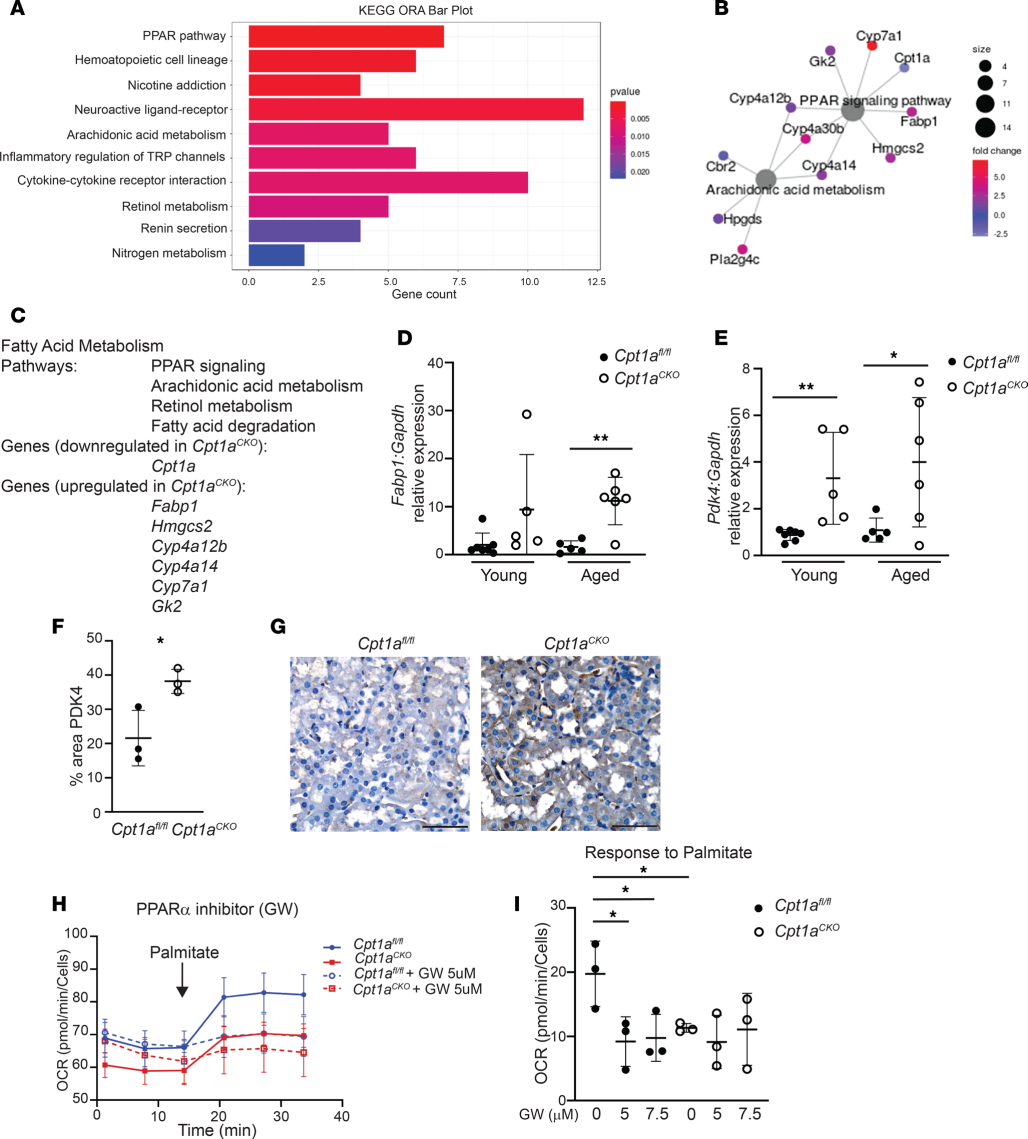
RNA-Seq showing *Cpt1a^CKO^* kidneys with upregulated PPAR gene expression. Bulk RNA-Seq on aged *Cpt1a^fl/fl^* and *Cpt1a^CKO^* kidneys and gene ontology overrepresentation analysis (ORA) were performed using enrichGO from clusterProfiler. Gene networks and pathways significantly altered between genotypes are shown (**A** and **B**), with the pathways and genes specifically related to fatty acid metabolism listed (**C**). Significant changes in *Fabp1*, a known transcriptional target of PPARα, expression, that were identified by RNA-Seq were confirmed with qPCR (*n* = 5–6) (**D**). *Pdk4*, another target involved in glycolysis, was significantly upregulated in aged *Cpt1a^CKO^* murine kidneys in RNA-Seq (**E**) and confirmed by qPCR (*n* = 5–6) and protein expression (*n* = 3) (**F** and **G**). Scale bar: 50 μm. Primary PT cells with or without PPARα inhibitor (GW6371) had OCR responses to palmitate measured by Seahorse with a representative tracing shown (**H**) and quantified (**I**). Data are shown as the mean ± SD. *P < 0.05, **P < 0.01. Statistical significance between the 2 genotypes was determined by unpaired *t* test for **D**–**F**. One-way ANOVA was performed followed by Šidák’s multiple comparisons test for **H**.

Deleting tubular CPT1A did not significantly affect aging or response to CKD models despite robust recombination, increased lipid droplet accumulation, and reduced FAO by bioflux analysis. Furthermore, tubular cells were still able to metabolize LCFA despite the absence of CPT1A. These findings raised two important questions: (a) why do kidneys with reduced FAO and increased lipid accumulation have such a mild phenotype after aging or injury, and (b) how are tubules lacking CPT1A still able to metabolize LCFA (at a reduced rate)? To address the first question, we hypothesized that compensatory changes may protect the *Cpt1a^CKO^* mice. To further explore, we performed RNA-Seq on aged renal cortices. A total of 302 genes were significantly upregulated and 35 genes downregulated in aged *Cpt1a^CKO^* mice compared with aged floxed controls. Several pathways involved in fatty acid metabolism were upregulated, including PPAR, arachidonic acid, and retinol metabolism (Figure 8, A–C). Many of the upregulated genes in aged *Cpt1a^CKO^* mice (e.g., *Fabp1*, *Hmgcs2*) are linked specifically to PPARα, an important stimulator of fatty acid metabolism. We confirmed with qPCR that *Fabp1* is upregulated in aged *Cpt1a^CKO^* mice (Figure 8D). Fabp1 encodes fatty acid binding protein 1 (FABP1), also known as L-FABP, which is restricted to the renal tubules (26); its expression is induced by both PPARα agonists and LCFA (27). Activation of PPARα has been shown by others to be protective in the aging kidney (9), and both PPARα activation and overexpression of FABP1 are protective in cisplatin and ischemia/reperfusion injuries, respectively (27). Thus, upregulated PPARα activity could potentially offset some of the detrimental effects of *Cpt1a* tubular deletion. Another target identified by RNA-Seq may explain why *Cpt1a^CKO^* mice have increased glycolytic capacity and reserve. *Pdk4*, encoding pyruvate dehydrogenase kinase 4 (PDK4), is upregulated in *Cpt1a^CKO^* mice as shown by RNA-Seq, and this increase was validated with increased gene and protein expression in *Cpt1a^CKO^* murine kidneys compared with those of floxed controls (Figure 8, E–G). PDK4 inhibits pyruvate oxidation by mitochondria through inhibitory phosphorylation of pyruvate dehydrogenase, thus diverting pyruvate to lactate production. This increased PDK4 expression could explain the greater glycolytic capacity in primary cells from *Cpt1a^CKO^* mice (Figure 6, H–J). Future studies are needed to confirm whether the increased glycolytic capacity is mediated by PDK4.

We next explored the question of how *Cpt1a^–/–^* tubular cells are able to oxidize LCFA, albeit at a reduced rate. CPT1A is the most abundant isoform in the kidney, but it is possible that upregulation of CPT1B, normally expressed in the skeletal muscle, heart, and adipose tissue, or CPT1C, usually expressed in the brain and testes, could compensate for the absence of CPT1A (11). However, data from RNA-Seq showed minimal gene expression of both isoforms in kidneys from both genotypes (Supplemental Figure 4). Consistent with this, CPT1B protein expression was not upregulated in the aged *Cpt1a^CKO^* mice or those injured by AAN or UUO (Supplemental Figure 4). Unlike CPT1A/B, CPT1C localizes to the endoplasmic reticulum not mitochondria and is unable to oxidize fatty acids (28, 29). Nonetheless, we measured CPT1C expression by immunohistochemistry, using brain as a positive control and found no expression in either the aged or injured murine kidneys of either genotype (Supplemental Figure 4). Given the lack of gene expression and protein expression of CPT1B/C, other CPT1 isoforms are unlikely to mediate FAO in tubules lacking CPT1A.

PPARα stimulates FAO by both mitochondria and peroxisomes, so we used a PPARα inhibitor to investigate whether this pathway was mediating FAO in the *Cpt1a^CKO^* tubules. Primary PT cells with CPT1A intact had a significant response to the PPARα inhibitor, but no response was observed in PT cells lacking CPT1A (Figure 8, H and I). It is possible that the PPARα inhibitor was blocking mitochondrial FAO (CPT1A is a downstream target), thus equalizing the response to palmitate between genotypes. However, PPARα activity is not responsible for the compensatory LCFA oxidation by PT cells lacking CPT1A.

**Figure 9:**
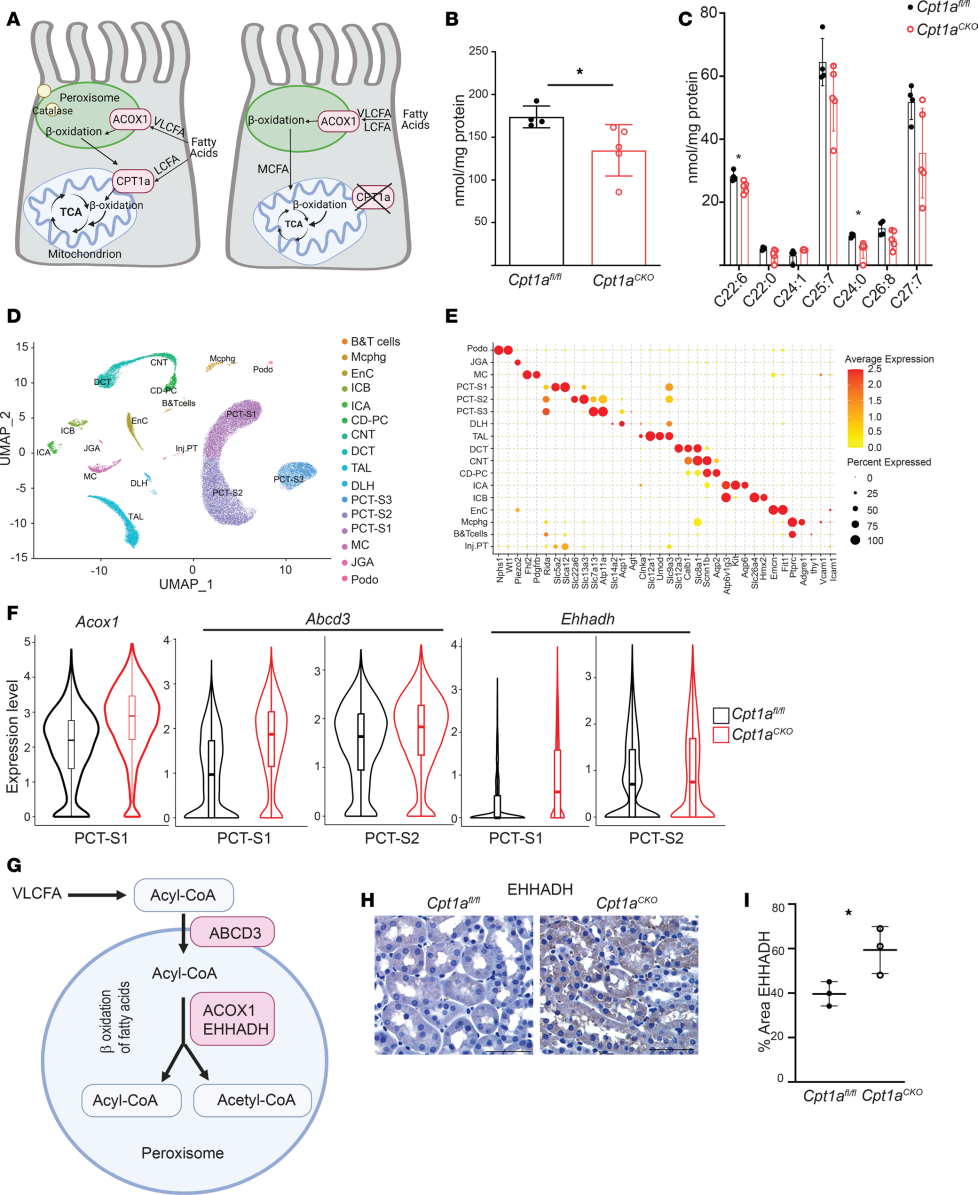
Peroxisomes likely compensate to metabolize LCFA in absence of CPT1A. CPT1A and ACOX1 regulate β-oxidation of LCFA and VLFCA, respectively, in mitochondria and peroxisomes. Schematic of how peroxisomes may compensate to metabolize LCFA through ACOX1 in the absence of CPT1A (**A**). (**B**) Total levels of VLCFA (≥22 carbons) and individual VLCFA (**C**) were analyzed using mass spectroscopy in kidney tissue from *Cpt1a^CKO^* mice and floxed controls (*n* = 4–5). Data are shown as the mean ± SD. *P < 0.05. Statistical significance between the 2 genotypes was determined by unpaired *t* test. UMAP projection of cell types derived from single-nucleus RNA-Seq of cortical kidney tissues isolated from 3-month-old *Cpt1a^fl/fl^* (*n* = 2) and *Cpt1a^CKO^* (*n* = 3) mice (**D**). A dot plot exhibiting expression levels of known, cell-type-specific marker genes and the percentage of cells in each cluster expressing cell-type-specific marker genes (**E**). Violin plots of peroxisomal genes significantly upregulated in segments of the proximal tubule with adjusted *P* values (*P < 0.01, **P < 1 × 10^–10^, ***P < 1 × 10^–20^; see Table 1 for specific *P* values and fold changes) (**F**). ABCD3 is a key transporter for importing VLCFA and LCFA into peroxisomes, and ACOX1 and EHHADH are enzymes involved in the first and second steps of peroxisomal FAO, respectively (**G**). Immunohistochemistry of EHHADH (**H**) and quantification (**I**) of young murine kidneys from *Cpt1a^fl/fl^* and *Cpt1a^CKO^* mice. Scale bar: 50 μm. VLCFA, very long-chain fatty acid; LCFA, long-chain fatty acid; ACOX1, acyl-CoA oxidase 1; TCA, tricarboxylic acid; PCT-S1, proximal tubule, segment S1; PCT-S2, proximal tubule, segment S2; EHHADH, enoyl-CoA hydratase and 3-hydroxyacyl CoA dehydrogenase.

Another potential pathway whereby Cpt1a-deficient tubule cells may oxidize LCFA is through peroxisomes, organelles that usually metabolize very-long-chain fatty acids (VLCFA) (Figure 9A). Others have shown that when the mitochondrial carnitine shuttle (CPT1/CPT2) is impaired in cells in vitro, peroxisomes compensate by oxidizing LCFA into medium-chain fatty acids, which are able to enter the mitochondria without the need for CPT1A or the carnitine shuttle (Figure 9B) (30, 31). As peroxisomes are the only organelles that metabolize VLCFA (≥22 carbons), we analyzed VLCFA concentrations in the *Cpt1a^CKO^* murine kidneys as a reflection of peroxisomal activity. Consistent with increased peroxisomal activity, kidneys from *Cpt1a^CKO^* mice had lower levels of total VLCFA (Figure 9, C and D).

Peroxisomes are localized to proximal tubules, so bulk RNA-Seq may have missed PT-specific changes in transcripts of peroxisomal FAO enzymes thought to reflect enzyme activity (32, 33). Therefore, we performed single-nucleus RNA-Seq (sn-RNA-Seq) on 3-month-old mice. Unsupervised hierarchical clustering of individual nuclei isolated from cortical tissues defined 16 clusters with strong enrichment of known cell-type-specific marker genes (Figure 9, E and F). As expected, uniform manifold approximation and projection (UMAP) plots highlight the enrichment of peroxisomal genes in proximal tubule segments (Supplemental Figure 5). Differential gene expression analysis of proximal tubule segments revealed increased expression of *Acox1*, which encodes the rate-limiting enzyme for peroxisomal FAO, as well as *Ehhadh*, which is also involved in peroxisomal FAO (Figure 9, F and G, and Table 1) (32, 34). Both of these peroxisomal FAO genes were among the top 10 most upregulated transcripts in the S1 segment of the proximal tubule. The gene encoding transporter ABCD3, required for compensatory peroxisomal FAO of LCFA when the carnitine shuttle is impaired (30), was also significantly increased in PT segments (Figure 9, F and G, and Table 1). Validation of increased peroxisomal FAO enzyme expression at the protein level is shown using immunohistochemistry for EHHADH (Figure 9, H and I). Taken together, the increase in peroxisomal FAO enzyme gene expression coupled with decreased VLCFA concentrations strongly supports increased peroxisomal activity as a compensatory mechanism to metabolize LCFA in the absence of CPT1A.

## Discussion

Unexpectedly, renal injury and fibrosis were minimally affected in the conditional *Cpt1a*-knockout (*Cpt1a^CKO^*) mice despite robust recombination. Deleting Cpt1a partially, but not completely, impaired FAO in primary PT cells, suggesting that CPT1A is not required for FAO. The PPAR pathway was identified by bulk RNA-Seq as the most upregulated pathway in aged kidneys lacking tubular CPT1A. However, compensatory changes in PPARα were not responsible for the ability of tubules lacking CPT1A to oxidize LCFA. Peroxisomes, the only other organelle capable of FAO, can compensate for LCFA metabolism when CPT1 or CPT2 are blocked in vitro (30, 31). Consistent with this, *Cpt1a^CKO^* kidneys had decreased levels of VLCFA, exclusively metabolized by peroxisomes, and increased gene expression of peroxisomal FAO enzymes. Taken together, these findings suggest compensatory increased peroxisomal β-oxidation in *Cpt1a^CKO^* kidneys.

The aging kidney is associated with altered lipid metabolism and decreased expression of genes involved in mitochondrial function and metabolism (35, 36). The highly metabolic proximal tubule requires FAO to properly function, and CPT1A, the rate-limiting enzyme for FAO, is decreased in aging and injured kidneys (1, 35). Furthermore, recent data suggest that overexpressing tubular Cpt1a is protective in kidney injury, and this is associated with increased FAO and improved mitochondrial function (3). However, whether CPT1A is required to maintain healthy tubules during aging and injury was unclear. Surprisingly, when we deleted *Cpt1a* in adult kidney tubules, the mice had a very subtle aging phenotype and no difference in renal function or fibrosis after renal injury. How do we reconcile the protective actions of CPT1A in the literature with the relatively mild aging phenotype and lack of effect in two CKD models? It is possible that augmenting FAO is protective as recently reported (3), but CPT1A might not be required for FAO and compensatory mechanisms (e.g., peroxisomal FAO) may abrogate the deleterious effects of *Cpt1a* deletion and compromised FAO.

Aged *Cpt1a^CKO^* mice had reduced palmitate-dependent oxidation, increased histological tubular injury in the inner cortex, increased inflammation, and significantly more Oil Red O staining and triglyceride accumulation, suggesting lipid accumulation. The fact that *Cpt1a^CKO^* mice have increased intracellular lipid accumulation but not increased fibrosis is consistent with prior data showing that overexpression of the fatty acid transporter CD36 increased tubular lipids but not fibrosis (1). Increased lipid accumulation has been associated with tubular injury and inflammation (37, 38), so these changes in the aged *Cpt1a^CKO^* mice may be a result of increased intracellular lipids.

The ability of tubules from *Cpt1a^CKO^* mice to metabolize LCFA, albeit at a reduced rate, was unexpected, given the need for a properly functioning carnitine shuttle to import palmitate to the mitochondria. Markedly reduced CPT1A expression was confirmed by immunofluorescence (IF) (Figure 1G), immunoblots (Figure 1H), and gene expression from RNA-Seq (Supplemental Figure 4). There was no evidence by gene expression or protein levels of CPT1B or CPT1C compensating in the absence of CPT1A (Supplemental Figure 4). CPT1C is not localized to the mitochondria and is unable to oxidize LCFA (28, 29), making it unlikely to explain the retained ability of tubule cells lacking CPT1A to oxidize LCFA.

A likely explanation for how *CPT1A^–/–^* tubule cells oxidize LCFA is that peroxisomes compensate to oxidize LCFA into MCFA, which can then enter the mitochondria independent of CPT1A for further metabolism. Peroxisomes typically oxidize VLCFA rather than LCFA, but when the carnitine shuttle is impaired, peroxisomes can oxidize LCFA such as palmitate in vitro (30, 31). Some have even defined peroxisomal FAO as palmitate oxidation in the presence of the CPT1A inhibitor etomoxir (39). Several findings support a compensatory upregulation of peroxisomal activity in the *Cpt1a^CKO^* kidneys. First, RNA-Seq data revealed upregulated gene expression in the PPAR pathway and of PPARα target genes in the *Cpt1a^CKO^* kidneys. PPARα is a well-known, potent stimulator of FAO in both mitochondria and peroxisomes. More specifically, the gene for FABP1 (*Fabp1*) was significantly upregulated in *Cpt1a^CKO^* kidneys, and others have shown a connection between FABP1 and peroxisomal proliferation and β-oxidation (27, 40). Pharmacologic inhibition of CPT1 in hepatocytes in vitro was associated with both upregulation of peroxisomal β-oxidation and FABP1 expression (41). The increased *Fabp1* gene expression in *Cpt1a^CKO^* kidneys suggests stimuli for augmented peroxisome activity. In addition, the reduced levels of VLCFA, exclusively metabolized through peroxisomes, provide functional data supportive of increased peroxisomal activity in kidneys with tubular deletion of Cpt1a. In addition, gene expression of *Acox1*, encoding the rate-limiting enzyme for peroxisomal FAO, and *Ehhadh*, another peroxisomal FAO enzyme were among the top 10 most upregulated genes within the S1 segment of the proximal tubule. The gene for ABCD3 was also significantly upregulated, and this transporter was shown by others to be required for compensatory peroxisomal FAO of LCFA in the absence of a functional carnitine shuttle (i.e., CPT1/2) (30). This upregulated peroxisomal FAO gene expression likely did not appear on bulk RNA-Seq owing to the dilution by nonproximal tubule cells. Unlike mitochondrial metabolism, which is constitutive, peroxisomal FAO is inducible, and synthesis of FAO proteins correlates well with enzyme activity (32, 33). Taken together, the increased expression of genes in the PPARα pathway, reduced VLCFA, and increased gene expression of peroxisomal FAO enzymes in the *Cpt1a^CKO^* mice strongly suggest increased peroxisomal FAO.

In addition to changes in FAO, tubular cells from *Cpt1a^CKO^* mice also had increased glycolytic capacity. Increased PDK4, which blocks pyruvate oxidation, could account for this increased glycolytic capacity. *Pdk4* gene expression is stimulated by both increased levels of fatty acids and the PPARα pathway (42), and we have evidence supporting both of these stimuli in the *Cpt1a^CKO^* mice (Figure 1, I–K, and Figure 8, A–D). No major significant reductions in mitochondrial complex expression were observed in *Cpt1a^CKO^* mice, but changes in mitochondrial morphology were noted by EM. It is also possible that subtle mitochondrial impairment could also lead to increased glycolytic capacity, though no basal differences in glycolysis were observed. It is possible that a switch to a more glycolytic capacity may be related to the increased kidney weight of *Cpt1a^CKO^* mice (Supplemental Figure 1). Alternatively, increased PPARα activity has been linked to renal proximal tubular hypertrophy (43). Further understanding of the mechanism of increased kidney weight in *Cpt1a^CKO^* mice will be pursued in future studies.

The aged *Cpt1a^CKO^* kidneys demonstrated subtle tubular injury, but the mild phenotype was somewhat surprising given the reduced FAO and this association with injury. Increased PPARα activity has been shown to protect against age-related renal fibrosis (9, 44), so it is possible that the compensatory increase in gene expression in the PPAR pathway offsets some of the detrimental effects of *Cpt1a* deletion, whether dependent on peroxisomes or not. It is also possible that other pathways (e.g., retinol metabolism, arachidonic acid metabolism) identified by RNA-Seq that are upregulated in the aged *Cpt1a^CKO^* kidneys may contribute to the milder-than-expected phenotype. RNA-Seq data show increased gene expression in the PPAR pathway and of PPARα targets specifically, but this does not necessarily mean increased PPARα activity. We confirm that PPARα activity is not responsible for the ability of tubule cells lacking CPT1A to oxidize LCFA.

Another group reported that *Cpt1a* deletion in renal tubules exacerbated injury induced by folic acid (45). RNA-Seq identified several genes in the family of ATP-binding cassette (ABC) transporters (e.g., *Abcd4*, *Abcg5*) as well as solute carrier transporters that are significantly upregulated in the *Cpt1a^CKO^* murine kidneys. These transporters have been linked to renal handling of folic acid (46), so it is possible that *Cpt1a^CKO^* mice sustained greater injury in the folic acid model due to increased uptake of folic acid through these upregulated transporters. In the hepatocyte, both genetic and pharmacologic inhibition of Cpt1a was paradoxically beneficial through reduced endoplasmic reticulum stress (47), suggesting that augmenting CPT1A might not always have beneficial effects and could be problematic as a therapeutic approach.

FAO is an important energy source for proximal tubules, but the expression of CPT1A is widespread throughout the kidney tubules. In fact, the distal convoluted tubule has the greatest gene expression of *Cpt1a* (Figure 1, A and B). Consistent with this, tubules with the strongest CPT1A protein staining lacked LTL but stained for the distal tubule marker NCC (Figure 1, C and D). This finding calls into question whether the distal convoluted tubule utilizes FAO, in contrast to the conventional dogma that distal tubules are more glycolytic. Given the widespread tubular expression of CPT1A, we took the approach of deleting CPT1A throughout the tubule.

In summary, deletion of tubular CPT1A minimally affected aging and had no effect on two models of CKD. Consistent with this, patients with CPT1A deficiency do not have major kidney defects (some with renal tubular acidosis) (48). Additionally, we showed that CPT1A deletion impaired, but did not completely block, oxidation of LCFA, suggesting compensatory pathways for LCFA metabolism. RNA-Seq data showed upregulation of the PPAR pathway and PPARα targets specifically, but this pathway was not responsible for compensatory LCFA oxidation in *CPT1A^–/–^* PT cells. Our data suggest that peroxisomes may compensate to allow for LCFA oxidation in the absence of CPT1A, but confirmatory studies with genetic models are needed. While CPT1A has garnered much attention related to proximal tubular FAO, peroxisomal FAO may be more important than previously recognized, particularly when mitochondrial FAO is impaired.

## Methods

### Sex as a biological variable.

Only male mice were used as there are sex-specific differences in expression of certain proteins involved in metabolism (34).

### Animal aging and injury studies.

To generate mice lacking CPT1A in kidney tubules, *Cpt1a^fl/fl^* mice provided by Toren Finkel were crossed with the Pax8-rTTA;tetO-Cre mice (13), and all mice are on a mixed background. Recombination was induced by placing 5-week-old male mice on a doxycycline-containing diet for 3 weeks. For aging studies, mice were aged for 24 months and then sacrificed, with “young” mice controls being 8–14 weeks old. For AAN, 8- to 12-week-old mice were given 5.5 mg/kg aristolochic acid (MilliporeSigma, 9451) by i.p. injection 3 times per week for 2 weeks (total 6 injections) and sacrificed 6 weeks after the last injection. UUO was performed by exposing the right kidney through a dorsal incision and ligating the ureter just distal to the renal pelvis. Mice were sacrificed 7 days later.

### Injury score.

Tubular injury was scored by colleagues in pathology, unaware of the treatment status of the mice. All cortical ×400 fields were scored as follows: 0, no injury; 1, 1%–25%; 2, 26%–50%; 3, 51%–75%; and 4, 76%–100%. Tubular injury was defined as protein cast formation, tubular dilatation, tubular cell swelling, vacuolization, or tubular cell degeneration. Glomerular injury (glomerulomegaly, mesangial expansion, hypercellularity, adhesions to Bowman’s capsule, endocapillary hypercellularity and hyaline deposits) were assessed but not quantified, as no significant injury was noted.

### Immunohistochemistry, staining, and EM.

Paraformaldehyde-fixed (PAFA-fixed), paraffin-embedded tissues were rehydrated, and antigen target retrieval was performed with 100 mM Tris buffer (pH 10) for immunohistochemistry or citrate buffer (Biogenex) for IF in a pressure cooker. The following primary antibodies were used: CPT1A (Abcam, ab128568), F4/80 (Abcam, ab6640), collagen I (Abcam, 34710), LTL-biotin (Vector Laboratories, B-1325), Aquaporin 2 (Novus, NB110-74682), NaCl Cotransporter (NCC, MilliporeSigma, AB3553), and Na-K-Cl Cotransporter (Thermo Fisher Scientific, 18970-1-AP). For immunohistochemistry, tissues were incubated with the appropriate biotinylated secondary antibody (Vector Laboratories), amplified with ABC Elite peroxidase (Vector Laboratories), and detected by DAB (MilliporeSigma, D4293). For IF, tissues were incubated with the appropriate secondary antibody and mounted in Prolong Gold with DAPI (Invitrogen, P36941). Rehydrated paraffin-embedded slides were incubated overnight with Picrosirius red (MilliporeSigma, 36-554-8), briefly washed in acetic acid, and dehydrated. Uninjured tissue and tissue without the primary antibody were used as negative controls. Images were taken with either a Nikon Eclipse E600 or Nikon Eclipse Ti2 microscope.

For Oil Red O staining, kidney tissue was fixed in 4% PAFA for 3 hours at room temperature, following by overnight incubation in 30% sucrose at 4°C, washed, and embedded in OCT media. Frozen sections (5 μM) were cut by cryostat, fixed with PAFA for additional 15 minutes followed by 60% isopropanol, and incubated with Oil Red O (saturated in isopropanol then diluted to 60%, MilliporeSigma). After staining, slides were dipped in 60% isopropanol and counterstained with Meyer’s hematoxylin. For quantification of staining, images of 10 fields were quantified using ImageJ (NIH), and both image acquisition and quantification were done without knowledge of the tissue’s genotype.

For EM, kidney tissue was fixed in a PAFA/glutaraldehyde solution and then processed and sectioned by the Molecular Microbiology Imaging Facility at Washington University in St. Louis. Acquired images of mitochondria were analyzed using NIH-developed ImageJ software. Mitochondrial area and AR, derived from lengths of major and minor axes, were measured with an AR value of 1 indicating a circle, and higher values indicating more elongated structure (49).

### Renal function and proteinuria.

Blood from the tail vein was collected from injured mice prior to euthanasia, placed in heparinized tubes, and centrifuged, and the plasma supernatant was used with Thermo Infinity Urea Reagent to determine blood urea nitrogen levels. tGFR was assessed by measurements of FITC-sinistrin clearance as previously described (50, 51). Briefly, FITC-sinistrin (MediBeacon Gmbh) at 0.15 mg/mg body weight was injected, and the half-life of FITC-sinistrin was calculated with a transdermal device using a 3-compartment model with linear fit using MPD Studio software, and half-life was converted to tGFR (μL/min/100 gm body weight). Proteinuria was quantified using the Exocell Albuwell M Test Kit and Creatinine Companion.

### Immunoblots.

Kidney tissue was minced in lysate buffer (150 mM NaCl, 50 mM Tris-HCL, 1 mM EDTA, and 2% SDS plus phosphatase and protease inhibitors), sonicated, centrifuged, reduced with dithiothreitol, and quantified using the BCA protein assay (Thermo Fisher Scientific). Proteins were separated by SDS-PAGE, transferred to nitrocellulose membranes, and incubated with the following primary antibodies: CPT1A (Abcam, ab128568), CPT1B (Santa Cruz, sc-20670), VDAC (Abcam, ab154856), p16 (Abcam, ab211542), GAPDH (Santa Cruz, sc-25778), and β-actin (Cell Signaling, 3700). Appropriate HRP-conjugated secondaries were used followed by ECL, and bands were detected by GeneGnome (Syngene) and quantified by ImageJ.

### qPCR.

RNA was extracted from cortical tissue and cells using the QIAGEN RNeasy Mini Kit, and Bio-Rad’s iScript cDNA Synthesis Kit generated cDNA. RNA was isolated from renal cortices that were mechanically disrupted in Lysis Matrix Tubes (MP Biomedicals) containing the lysis buffer (RLT) supplemented with 1% β-mercaptoethanol before being purified on the silica membrane RNeasy spin columns. Quantitative real-time PCR was performed with 25–100 ng cDNA and iQ SYBR Green Supermix using the Bio-Rad CFX96 thermal cycler. Relative mRNA expressions were determined by the ΔΔCT equation, and Gapdh was used as a reference gene. The primers used are listed in Supplemental Table 1.

### Generation of primary proximal tubule–enriched cells for measurement of oxygen consumption.

Proximal tubule–enriched cell populations were generated as previously described (21) and characterized (20). Briefly, kidney cortical tissue was finely minced and digested in collagenase II (1 mg/mL), dispase (1 mg/mL), DNAse (1:100) in RPMI for 45 minutes at 37°C in a shaker. The slurry was strained with a 70 μM filter, washed with RPMI and centrifuged 3 times, and plated in RPMI 1640 with 10% FBS, 1 mM glucose, penicillin/streptomycin, and supplements (hydrocortisone, insulin, transferrin, selenium, and triiodothyronine, ascorbate). After the first 2 days, ascorbate was removed and FBS was reduced to 5%.

### Bioflux analysis.

Four days after initial plating, primary cells were replated on 24-well plates (100,000 cells per well) to be analyzed the following day using a Seahorse XF24. For measurement of fatty acid–dependent OCR, cells were plated in a low-glucose KHB media (111 mM NaCl, 4.7 mM KCl, 1.25 mM CaCl_2_, 2 mM MgSO_4_, 1.2 mM NaH_2_PO_4_, glucose 0.5 mM, 0.5 mM carnitine, and 5 mM HEPES with pH adjusted to 7.4). Glycolysis was measured separately using cells plated in Seahorse XF Base Medium with 1 mM glutamine, pH 7.4, and extracellular acidification rate was assessed at baseline, after addition of glucose, then 2-deoxyglucose, and antimycin/rotenone. Protein amounts were quantified using the SRB assay (BioVision). Analyses on young mice were done using Seahorse XF96, so cells were plated on a 96-well plate (63,000 per well). OCR was measured at baseline and after the injections of palmitate/BSA (175 μM, oligomycin, 3 μM), FCCP (2 μM), and antimycin/rotenone (2 μM). GW66471 (5 μM, S2798 Selleckchem) was used as PPARα inhibitor. After Seahorse analyses, the OCR was normalized to number of cells determined after methanol fixation, DAPI staining, and counting with Lionheart FX imager (Biotek).

### Measurement of F2-isoprostanes.

Urinary F2-isoprostanes were measured by Vanderbilt University Medical Center’s Eicosanoid Core Laboratory using gas chromatography/negative ion chemical ionization mass spectrometry and normalized by creatinine.

### Mitochondrial DNA copy number and common mutation.

Nuclear and mitochondrial DNA were isolated using a DNeasy kit (Qiagen), and the mitochondrial gene *ND1* was quantified by qPCR and normalized to the nuclear-encoded actin to measure mitochondrial DNA copy number. To reflect mitochondrial DNA mutations, we used the common *D17* mutation as previously described (25) and normalized to mitochondrial gene expression (ND1).

### Bulk RNA-Seq.

RNA was isolated from cortical tissue as described in *qPCR*, and the Illumina TruSeq stranded mRNA kit was used to generate a library prep with the NovaSeq sequencer and an average read length of 100 base pairs. Alignment was performed using STAR (v2.7.3a) aligner, with the raw read counts estimated using HTSeq and normalized using DESeq2. The aligned reads were used for estimating expression of the genes using cufflinks (v2.2.1), and expression values were reported in fragments per kilobase per million. Data quality check was performed using FastQC (https://www.bioinformatics.babraham.ac.uk/projects/fastqc/) with low-quality sequence reads excluded from analysis. Overrepresentation analysis, gene set enrichment analysis, and pathway analysis was performed for significantly differentially expressed protein coding genes. Database for Annotation, Visualization and Integrated Discovery (DAVID; https://david.ncifcrf.gov) v6.8 and Kyoto Encyclopedia of Genes and Genomes (KEGG; https://www.genome.jp/kegg/) were used for pathway analysis. Overrepresentation analysis and gene set enrichment analysis were performed using an R bioconductor package clusterProfiler (v3.13.4). Medgenome was used to carry out both the RNA-Seq and data analysis.

### Fatty acid extraction and mass spectrometric analysis.

Kidney tissue samples were homogenized in ice-cold PBS, and fatty acids were extracted as previously described (52). Briefly, 50 mg protein kidney homogenates containing 60 pmol internal standard (docosanoic-22,22,22-D_3_ acid) were transferred to glass tubes and subjected to complete hydrolysis using acid hydrolysis reagent (CH_3_CN/37% HCl, 4:1) followed by extraction of fatty acids using hexane. The isolated fatty acids were analyzed by electrospray ionization mass spectrometry using a Thermo LTQ Orbitrap Velos mass spectrometer, operated using Xcalibur software (Thermo Fisher Scientific).

### Tissue triglyceride measurements.

Tissue TAG levels were measured using Triglycerides Reagent (Thermo Fisher Scientific, TR22421) and Triglyceride Standard (Pointe Scientific, T7531STD) according to a modified protocol (53). In brief, tissue samples were homogenized in PBS (1/30, w/v). Then, 2.5 μL of the standard or sample homogenization and 250 μL of the reagent were added to each microplate well. After incubation at 37°C for 10 minutes, absorbances at 500 nm were measured using a plate reader from Tecan.

### High-resolution respirometry on kidney tissue ex vivo.

Small pieces of cortical tissue (1–3 mg wet weight) from kidneys from young mice (2–4 months old) were dissected in BIOPS (54) and washed twice in Buffer Z (55). Oxygen consumption was measured in Buffer Z at 37°C (Oxygraph O2k, Oroboros Instruments) in the presence of fatty acid substrate and tricarboxylic acid intermediates (0.5 mmol/L malate, 2 mmol/L L-carnitine, 20 μM palmitoyl-CoA, 2 mmol/L ADP, 10 mmol/L succinate).

### Single-nuclear RNA-Seq and informatics.

Cortical tissues were immediately snap frozen and stored in liquid nitrogen until single-nucleus suspension preparation. Tissues were thawed, minced, and homogenized in Nuclei EZ lysis buffer (MilliporeSigma) with a mortar and pestle (DKW) as previously described (16). Nuclei were counted and barcoded with a 10X Genomics’ 3′ v3.1 kit and protocol at 10,000 cells per sample, according to the manufacturer’s instructions. Libraries were constructed with 10X Genomics’ library preparation kit and sequenced on a NovaSeq6000 (Illumina) at up to 50 million reads per cell at Washington University’s Genome Technology Access Center. Count matrices produced for each sample with the CellRanger package were processed in the SoupX package (v. 1.4.2) to remove ambient background signals (56), using a contamination rate of 0.3, and then filtered for cell-cell doublets twice with the DoubletFinder package (v. 2.0.3) (57). Samples were then merged into a single object and filtered to include only cells containing 750–5,000 genes per cell, 12,500 maximum transcripts per cell, less than 0.5% mitochondrial gene content, 0.05% hemoglobin gene content, and 5% ribosomal subunit-associated gene content. All mitochondrial and hemoglobin genes were deleted after inclusion filters. Remaining cells were log transformed and scaled, with determination of variable features and PCA analysis using default settings in Seurat (v. 4.4.0) (58). The data were integrated with the Harmony package (v. 1.0.3) using samples as the covariate, with sigma = 0.07 and no early stop (59). Clusters were refined by removing additional doublets from each individual cluster, with doublet subclusters defined as low cell number clusters with distinct transcript and/or gene counts expressing marker genes associated with multiple cell types. The curated data set was finally clustered at a cluster resolution of 0.2 and visualized by UMAP using the harmony reduction at 26.5 dimensions in Seurat (v. 4.4.0). Up- and downregulated differential gene expression comparing Cpt1a^CKO^ to Cpt1a^fl/fl^ cells within each cluster was performed using the FindMarker function in Seurat, at default thresholds.

### Statistics.

Results are shown as the mean ± SD. GraphPad Prism9 was used to perform statistical analyses, with unpaired 2-tailed *t* test used to detect differences between 2 groups. For multiple comparisons, 1-way ANOVA was performed followed by Šidák’s multiple comparisons. The Wilcoxon’s rank-sum test was utilized by the FindMarker function in Seurat for differential gene expression analysis of sn-RNA-Seq data. *P* values of less than 0.05 were considered significant.

### Study approval.

All procedures were approved by the Institutional Animal Care and Use Committee of Vanderbilt University Medical Center and conducted according to the NIH *Guide for the Care and Use of Laboratory Animals* (National Academies Press, 2011).

### Data availability.

The raw data for bulk RNA-Seq has been deposited in GEO (GSE205497) to be publicly available upon publication. For sn-RNA-Seq, Fastq files and the main data object are available at GEO (GSE246150). Values for all data points in graphs are reported in the Supporting Data Values file.

## Author contributions

Both AI and SH conducted most of the experiments, including Seahorse analysis and analysis of injury, with AI leading the project at Vanderbilt and SH assuming the lead role at Washington University in St. Louis; both contributed to writing the Methods and figure legends. YO and J. Kern performed experiments. OV performed the UUO surgeries. RD and MDC performed the tGFR measurements. HY, AF, and NM did the tubule injury scoring and assessment of EM injury. DL and IJL performed the mass spectrometry analysis of fatty acids in kidney tissues. MPM, JT, and SC performed the staining, quantification, and animal husbandry. DW and LL performed the high-resolution respirometry. SF performed the sn-RNA-Seq studies with the assistance of J. Koenitzer and BDH. TF provided the Cpt1a-floxed mice. LG designed the experiments and wrote the manuscript; manuscript editing was done by TF, DW, MDC, IJL, AI, SC, LL, and DW.

## Supplementary Material

Supplemental data

Unedited blot and gel images

Supporting data values

## Figures and Tables

**Table 1:**
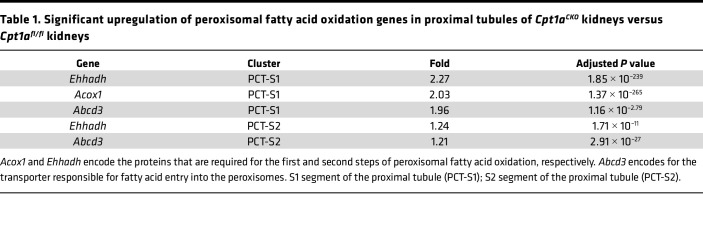
Significant upregulation of peroxisomal fatty acid oxidation genes in proximal tubules of *Cpt1a^CKO^* kidneys versus *Cpt1a^fl/fl^* kidneys

## Version 1 03/22/2024

Electronic publication

## Version 2 03/27/2024

David H Wasserman and Leslie S. Gewin's names were updated
