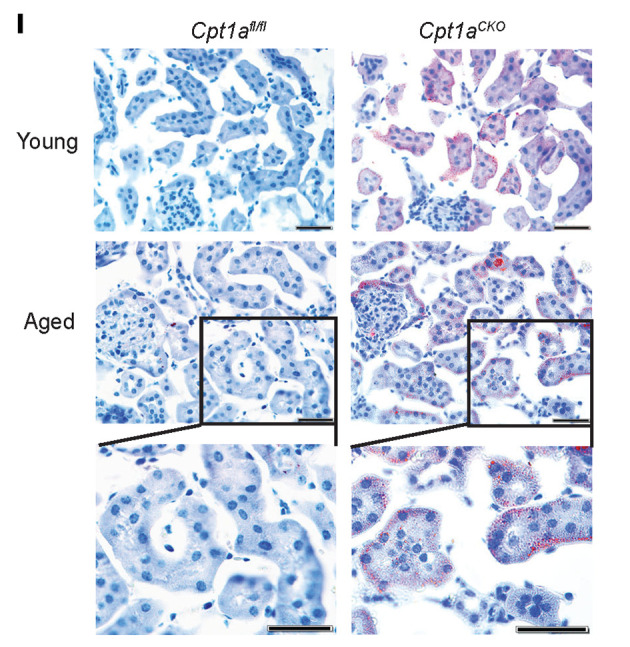# Tubular CPT1A deletion minimally affects aging and chronic kidney injury

**DOI:** 10.1172/jci.insight.181816

**Published:** 2024-05-08

**Authors:** Safaa Hammoud, Alla Ivanova, Yosuke Osaki, Steven Funk, Haichun Yang, Olga Viquez, Rachel Delgado, Dongliang Lu, Melanie Phillips Mignemi, Jane Tonello, Selene Colon, Louise Lantier, David H. Wasserman, Benjamin D. Humphreys, Jeffrey Koenitzer, Justin Kern, Mark de Caestecker, Toren Finkel, Agnes Fogo, Nidia Messias, Irfan J. Lodhi, Leslie S. Gewin

Original citation *JCI Insight*. 2024;9(6):e171961. https://doi.org/10.1172/jci.insight.171961

Citation for this corrigendum: *JCI Insight*. 2024;9(9):e181816. https://doi.org/10.1172/jci.insight.181816

The authors recently became aware of an inadvertent error in Figure 1I. The Young, *Cpt1a^CKO^* panel was incorrect and the same as the Aged, *Cpt1a^CKO^* panel. The correct panel is shown below.

The authors regret the error.

## Figures and Tables

**Figure 1:**