# Supplementary material for: Tubular CPT1A deletion minimally affects aging and chronic kidney injury
Source: JCI Insight. 2024 Mar 22;9(6):e171961. doi: 10.1172/jci.insight.171961 (PMC11063933; doi:10.1172/jci.insight.171961)
Supplement: Supplemental data [file jciinsight-9-171961-s286.pdf]

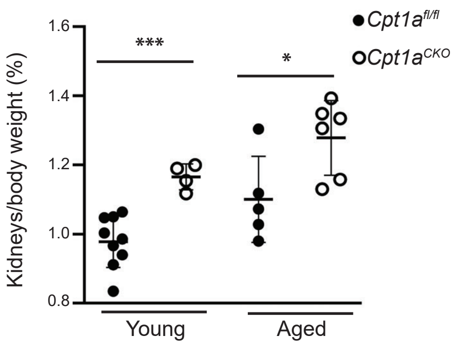

Supplemental Figure S1. Supplemental Figure S1. Cpt1aCKO mice have larger kidneys. Kidney weights expressed as a percentage of body weight in young and aged Cpt1afl/fl and Cpt1aCKO mice. Means are shown +/- SD with * = p<0.05 and ** = p<0.01.

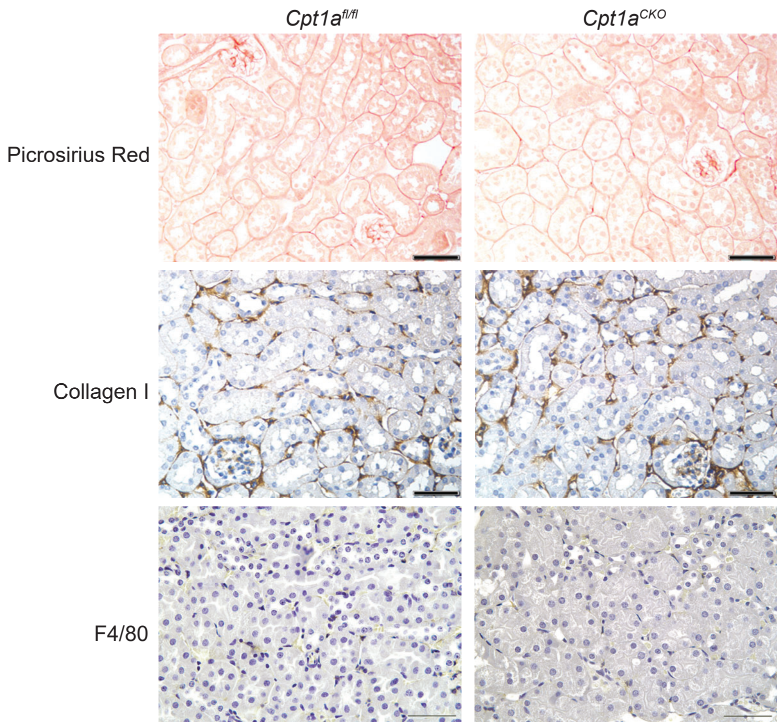

Supplemental Figure S2. Supplemental Figure S2. No difference in fibrosis or inflammation in young *Cpt1a*^{CKO} mice compared to floxed controls. Picrosirius Red staining, IHC of collagen I and F4/80 on kidney tissue of young *Cpt1a*$^{fl/fl}$ and *Cpt1a*^{CKO} mice. Scale bars = 50μM.

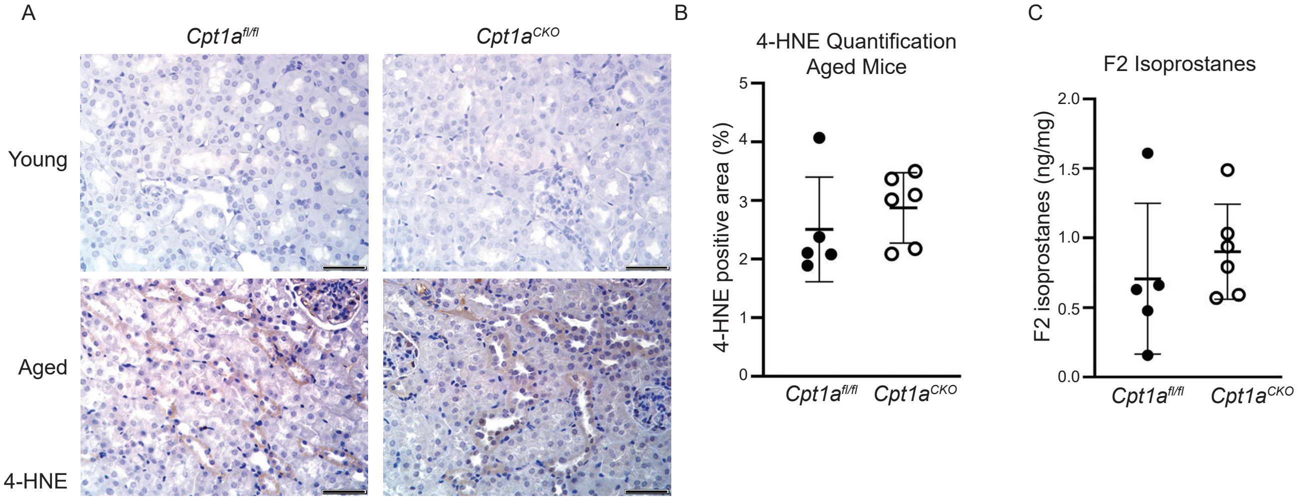

Supplemental Figure S3. No difference in 4-HNE or F2 isoprostanes between genotypes. 4-HNE staining was done on aged kidney tissues and quantified using ImageJ (A, B). Urine F2 isoprostanes were measured from aged mice and reported normalized to creatinine (C). Scale bars = 50μM.

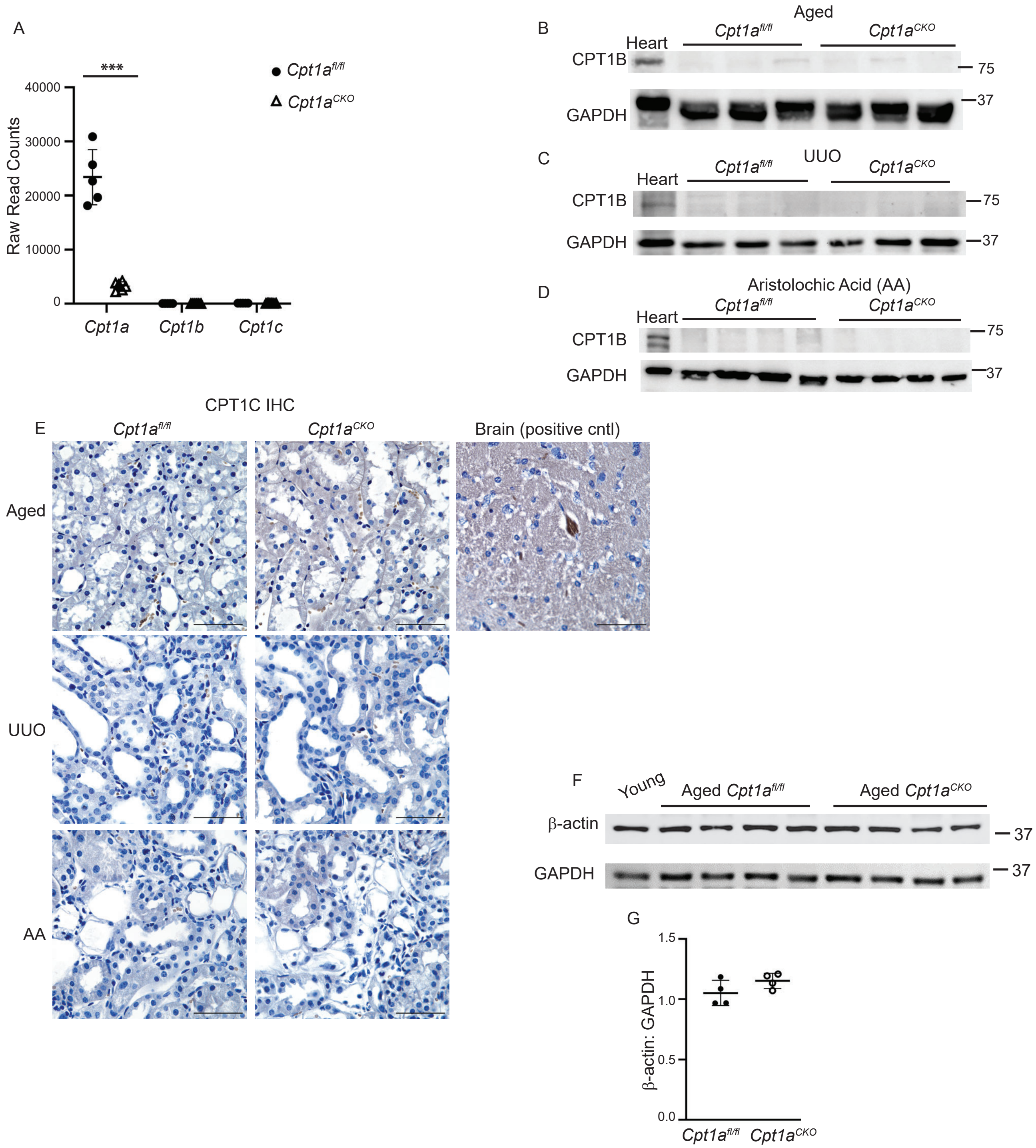

Supplemental Figure 4. Supplemental Figure S4. *Cpt1a*^{CKO} kidneys do not have compensatory upregulation of *Cpt1b* and *Cpt1c.*
(A) Transcript levels of *Cpt1a*, *Cpt1b*, and *Cpt1c* from RNAseq are shown for each genotype. There was only a statistical difference between genotypes in *Cpt1a* levels, means +/- SD shown, with p value calculated using the DESeq2 package. Immunoblots for CPT1B on kidney tissue lysates from aged (B), UUO-injured (C) or AAN-injured (D) mice with heart as a positive control.
(E) Immunohistochemistry for CPT1C with brain as positive control. As heart does not express β-actin, we used GAPDH for loading control and show that GAPDH and β-actin have no significant differences as loading controls when CPT1A is deleted (F, G).

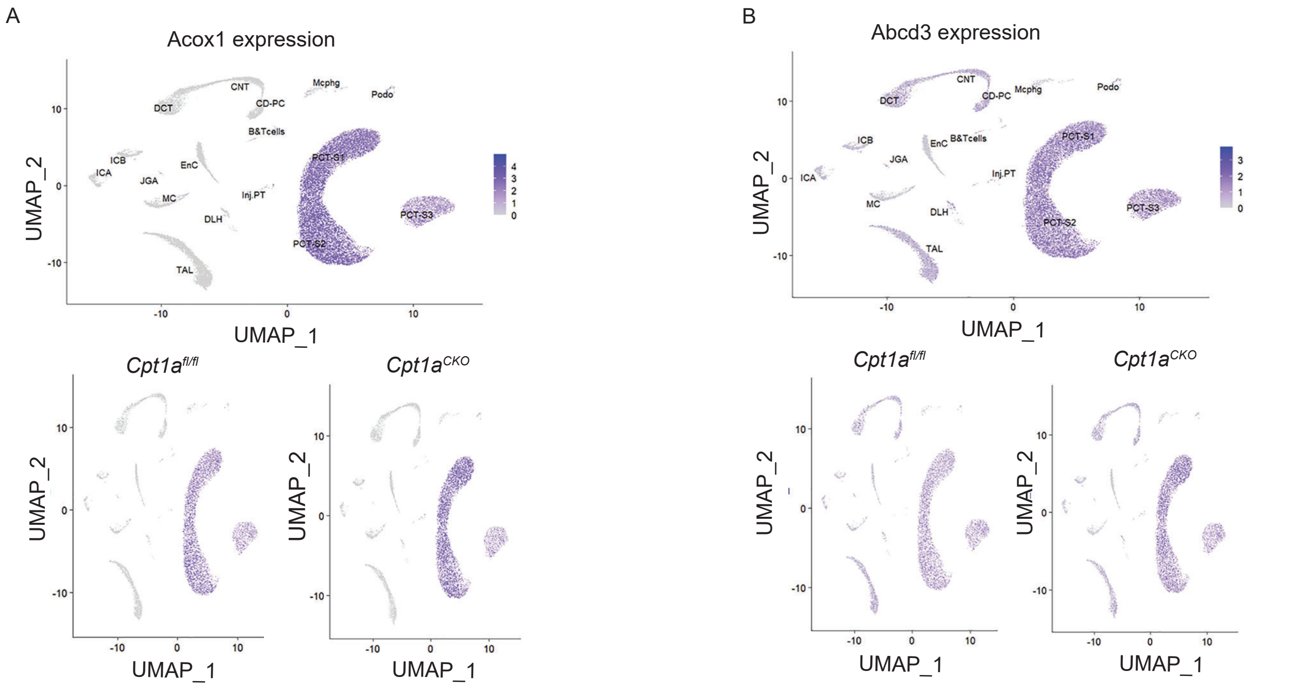

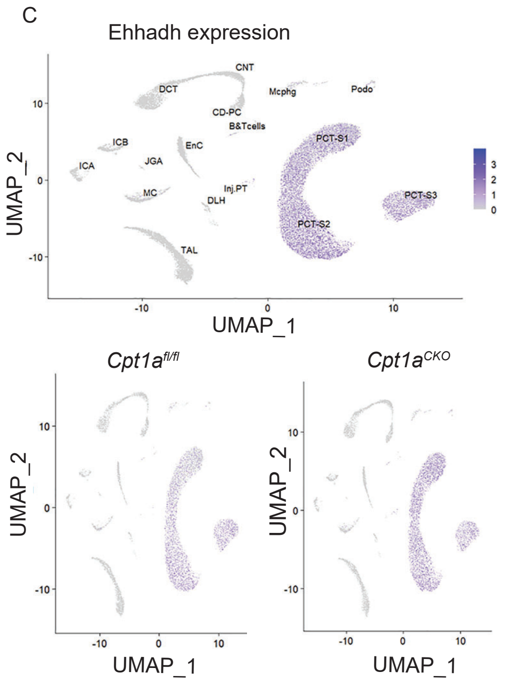

Supplemental Figure S5. UMAP dimension plots of peroxisomal genes that were significantly upregulated in *Cpt1a*^{CKO} mice. Expression of *Acox1* (A), *Abcd3* (B), and *Ehhadh* (C) in different clusters from single nuclear RNA-seq shows proximal tubule-specific enrichment and increased expression in *Cpt1a*^{CKO} versus *Cpt1a*$^{\text{fl/fl}}$ kidneys.

Table. List of primers used in the study

Gene name	Forward	Reverse
Acox1	5'-AGGGAATTTGGCATCGCAGA-3′	5′-CATGCCCAAGTGAAGGTCCA-3′
Actin	5'-GGGATGTTTGCTCCAACCAA-3′	5′-GCGCTTTTGACTCAGGATTTAA-3′
aSMA	5'-CAGGGAGTAATGGTTGGAAT-3′	5′-TCTCAAACATAATCTGGGTCA-3′
Col1a2	5'-GGAGGGAACGGTCCACGAT-3′	5′-GAGTCCGCGTATCCACAA-3′
D17 del	5'-GAACCAAACTGAACGCCTAAAC-3′	5′-TGGGCTTTTGGTAGTCATAGGT-3′
FABP1	5'-ATGAAGGCAATAGGTCTGCCC-3′	5′-CGATTTCTGACACCCCCTTGA3′
GAPDH	5′-AGGTCGGTGTGAACGGATTTG-3′	5′-TGTAGACCATGTAGTTGAGGTCA-3′
Havcr1 (Kim-1)	5′-AAACCAGAGATTCCCACACG-3′	5′-GTCGTGGGTCTTCCTGTAGC-3′
Hmgcs2	5'-CAGTGGAAGCAAGCTGGAAAC-3'	5′-TCTTGCAAAAGGGTGTGTGG-3′
IL1b	5'-CCCAAAAGATGAAGGGCTGC-3′	5′-TGATGTGCTGCTGCGAGATT-3′
IL6	5'-CTCTGCAAGAGACTTCCATCCA-3′	5′-AGTCTCCTCTCCGGACTTGT-3′
mtND1	5'-TAGAACGCAAAATCTTAGGG-3′	5′-TGCTAGTGTGAGTGATAGGG-3′
PDK4	5'-GCTGCTGGACTTTGGTTCAGA-3′	5'-GGATATTGGCCAGGCGGAC-3′
