# Supplementary material for: Tubular CPT1A deletion minimally affects aging and chronic kidney injury
Source: JCI Insight. 2024 Mar 22;9(6):e171961. doi: 10.1172/jci.insight.171961 (PMC11063933; doi:10.1172/jci.insight.171961)
Supplement: Unedited blot and gel images [file jciinsight-9-171961-s287.pptx]

## Slide 1
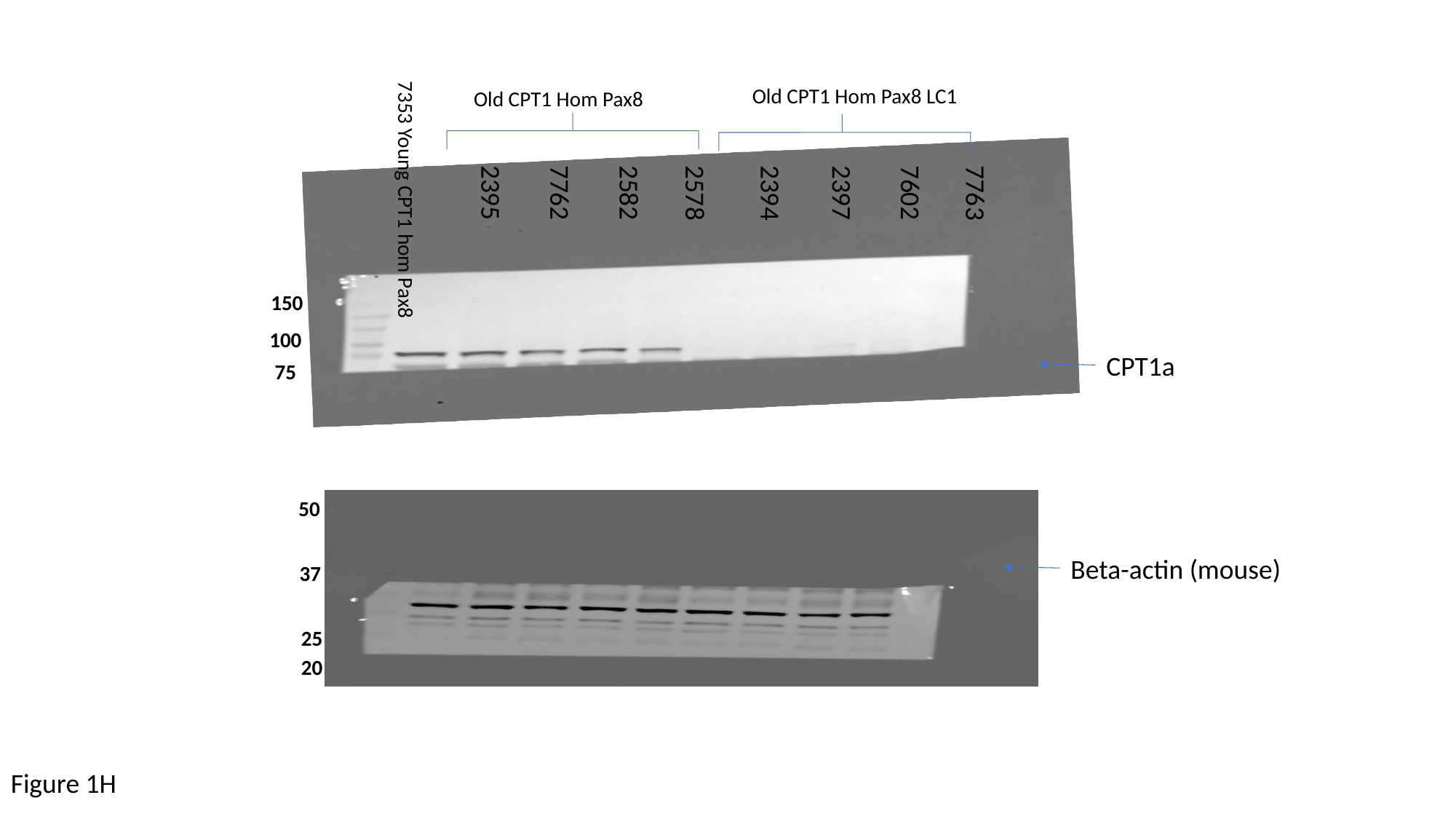

7353 Young CPT1 hom Pax8
Old CPT1 Hom Pax8 LC1
Old CPT1 Hom Pax8
2395
7762
2582
2578
2394
2397
7602
7763
150
100
CPT1a
75
50
Beta-actin (mouse)
37
25
20
Figure 1H

## Slide 2
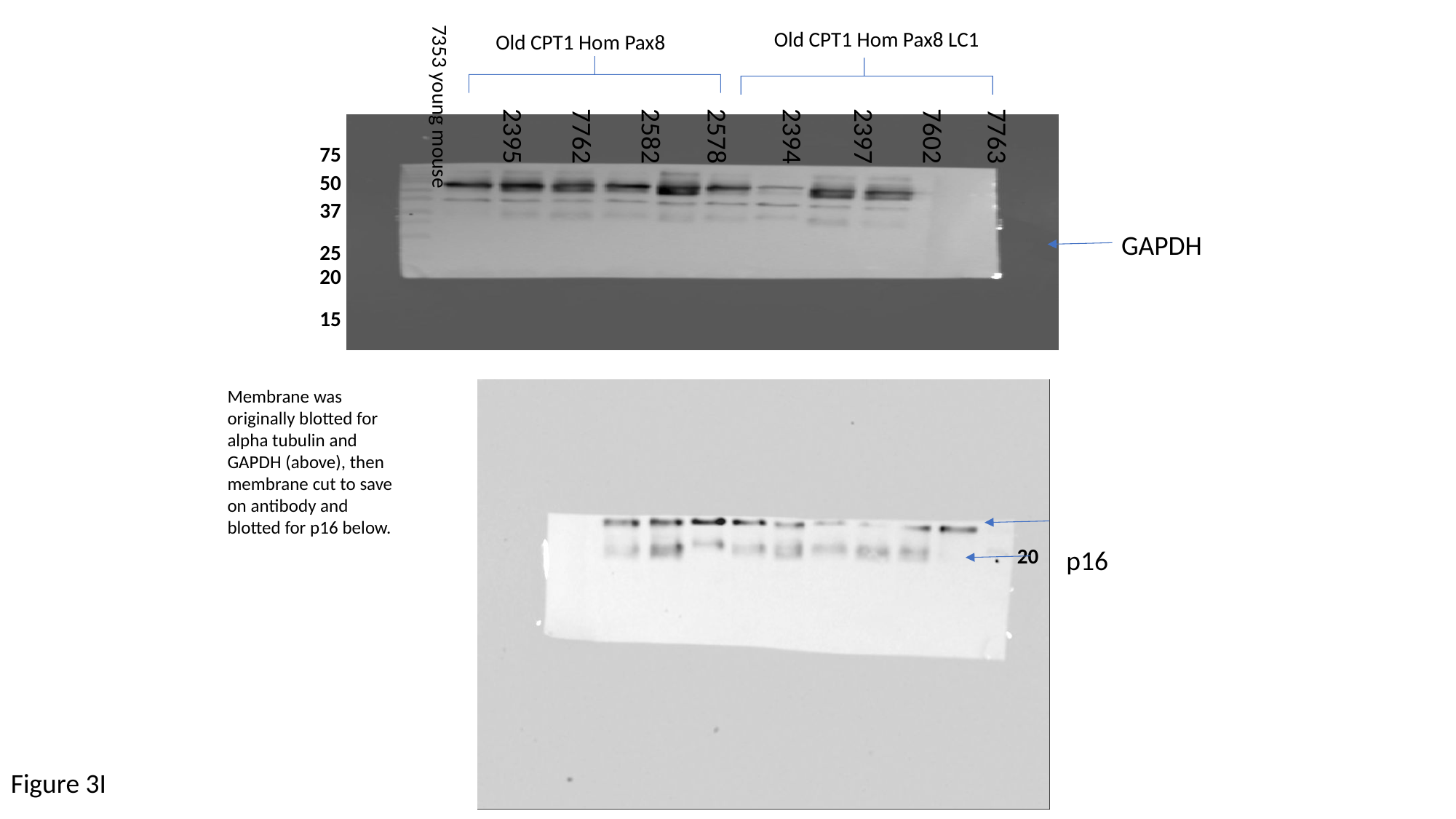

7353 young mouse
Old CPT1 Hom Pax8 LC1
Old CPT1 Hom Pax8
2395
2578
2394
7762
2582
2397
7602
7763
75
50
37
GAPDH
25
20
15
Membrane was originally blotted for alpha tubulin and GAPDH (above), then membrane cut to save on antibody and blotted for p16 below.
p16
20
Figure 3I

## Slide 3
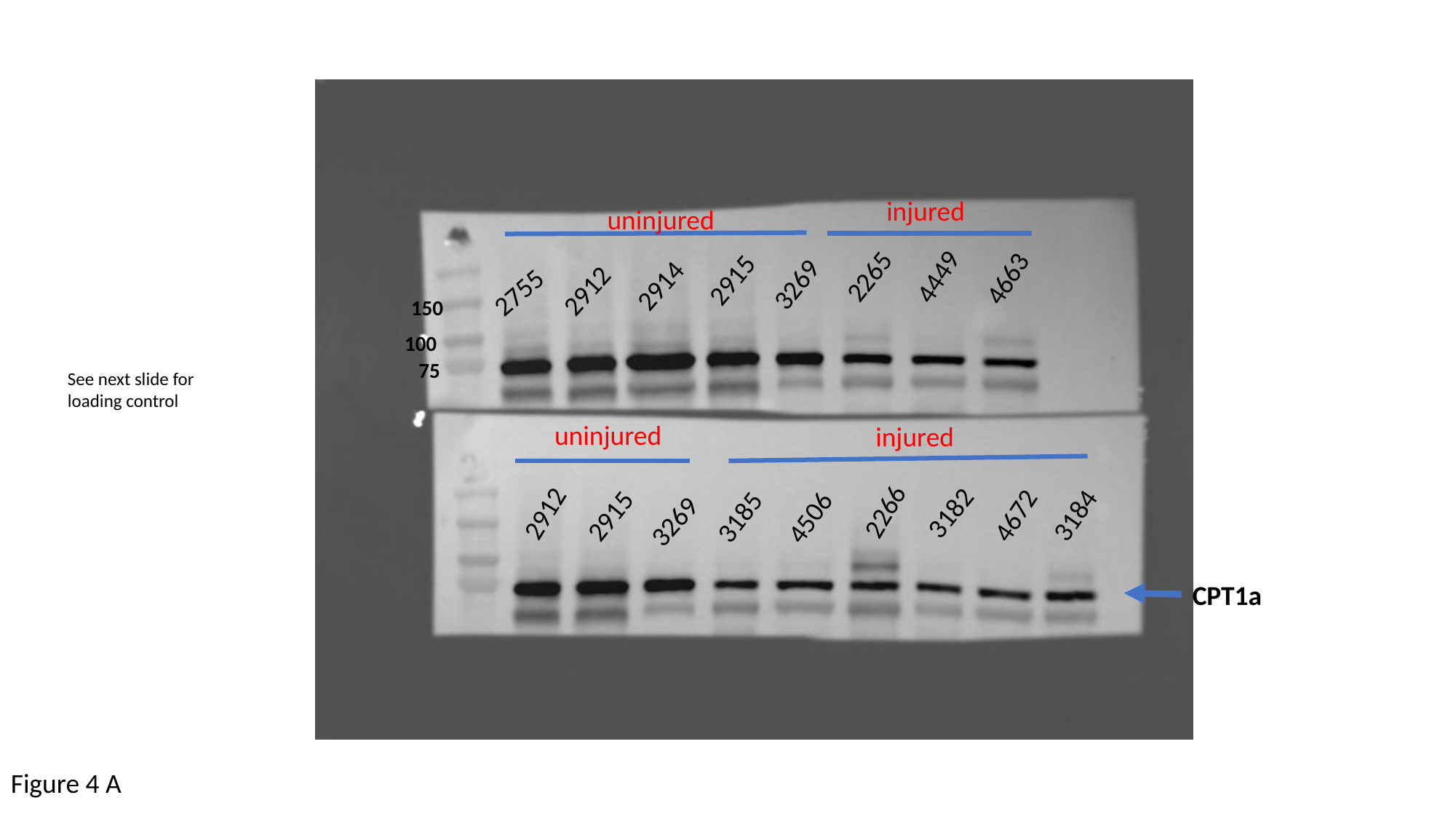

injured
uninjured
2915
2265
4449
3269
4663
2912
2914
2755
150
100
75
See next slide for loading control
uninjured
injured
2266
2912
4672
3184
2915
3185
4506
3182
3269
CPT1a
Figure 4 A

## Slide 4
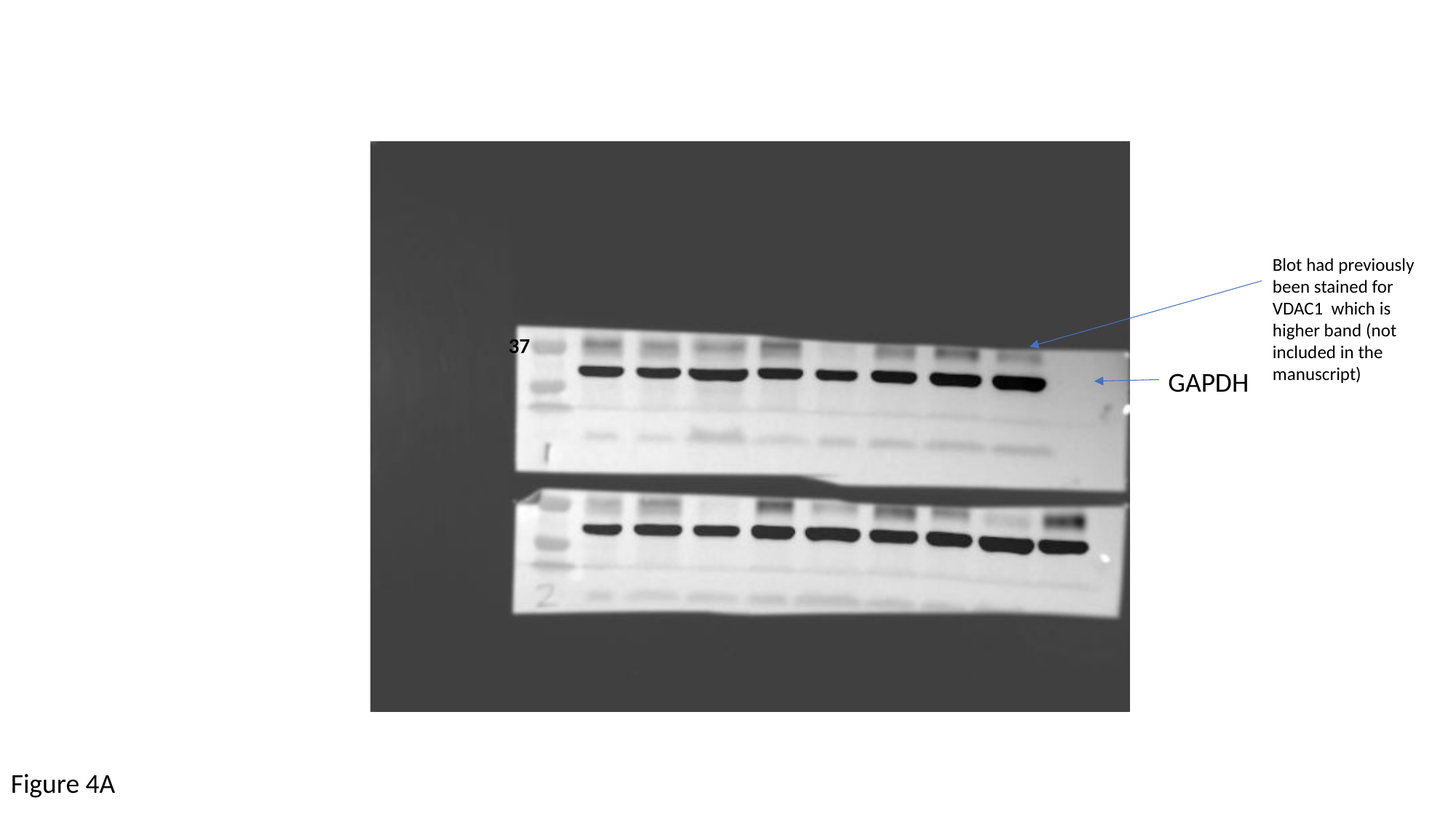

Blot had previously been stained for VDAC1 which is higher band (not included in the manuscript)
37
GAPDH
Figure 4A

## Slide 5
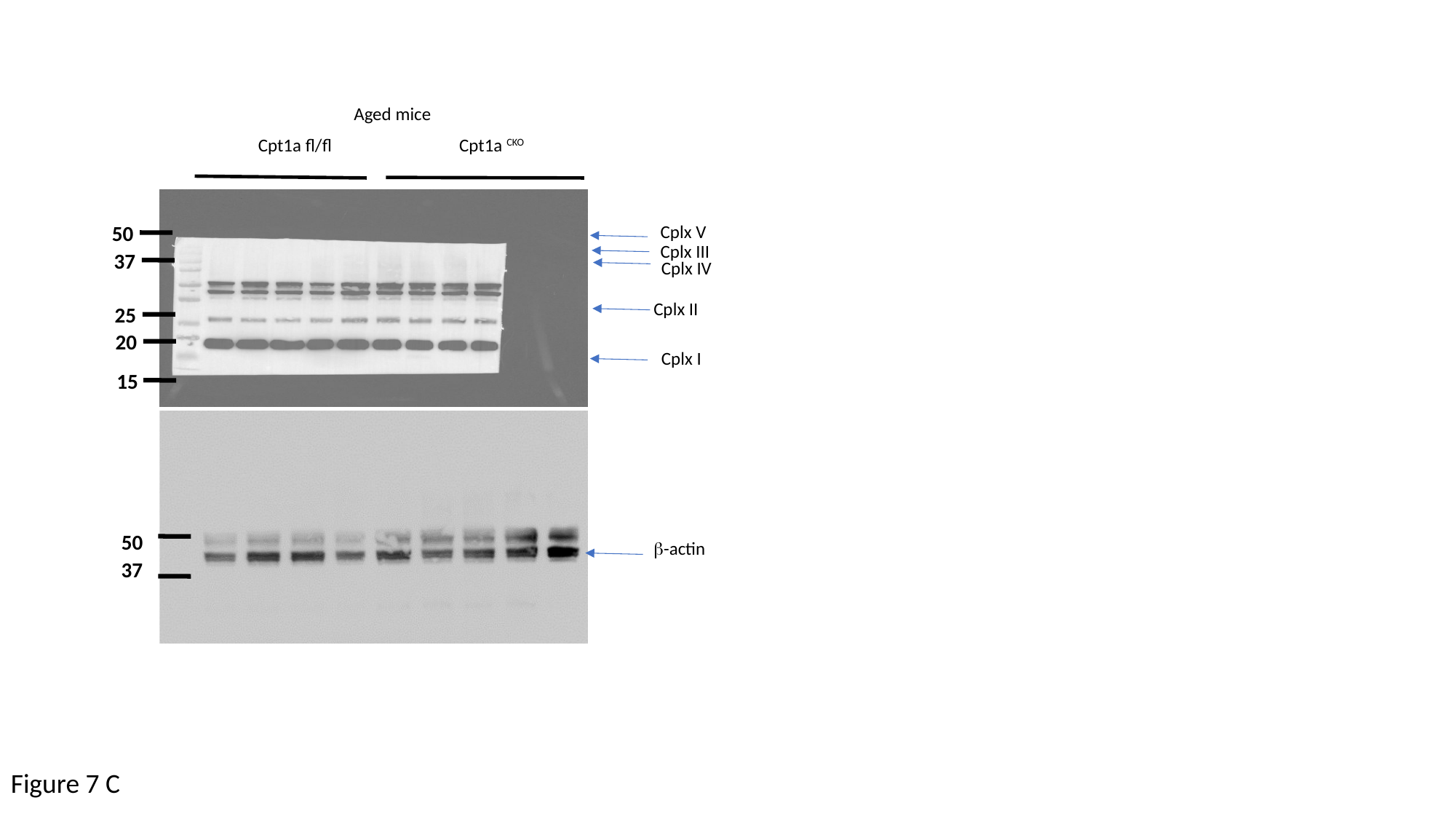

Aged mice
Cpt1a CKO
Cpt1a fl/fl
50
Cplx V
Cplx III
37
Cplx IV
Cplx II
25
20
Cplx I
15
50
b-actin
37
Figure 7 C

## Slide 6
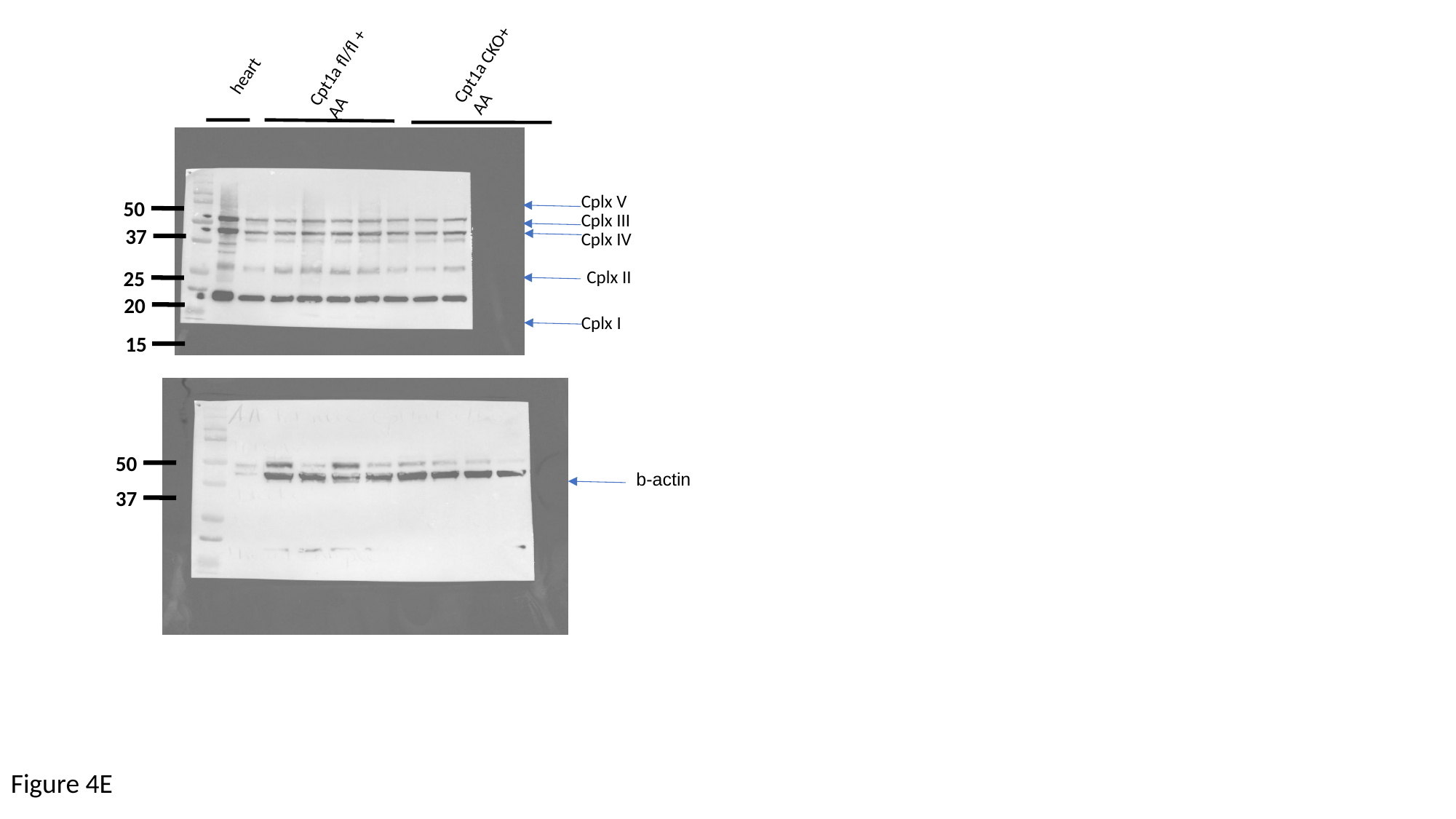

Cpt1a CKO+ AA
Cpt1a fl/fl + AA
heart
Cplx V
50
Cplx III
37
Cplx IV
25
Cplx II
20
Cplx I
15
50
b-actin
37
Figure 4E

## Slide 7
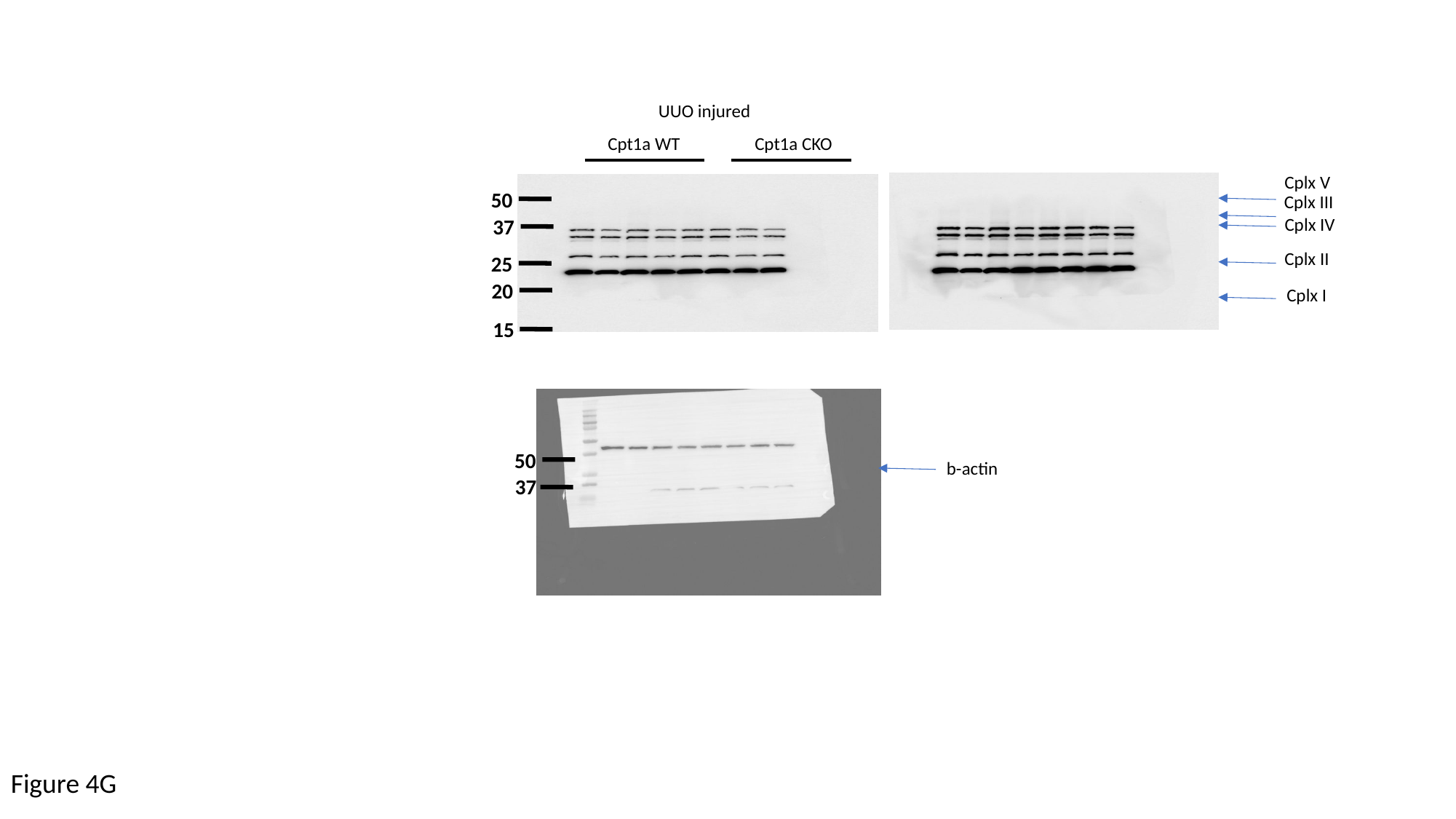

UUO injured
Cpt1a WT
Cpt1a CKO
Cplx V
50
Cplx III
Cplx IV
37
Cplx II
25
20
Cplx I
15
50
b-actin
37
Figure 4G

## Slide 8
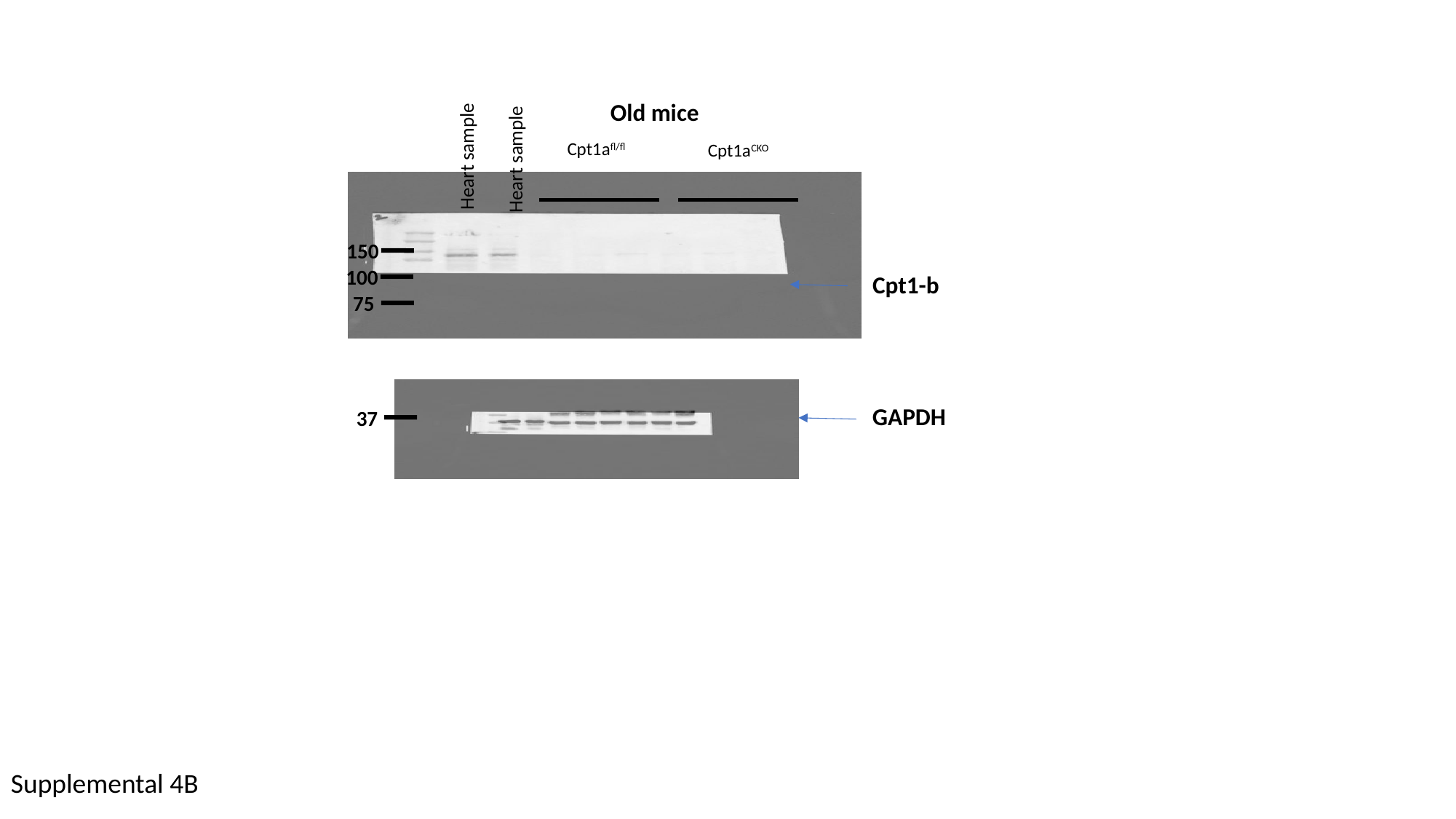

Old mice
Cpt1afl/fl
Cpt1aCKO
Heart sample
Heart sample
150
100
Cpt1-b
75
GAPDH
37
Supplemental 4B

## Slide 9
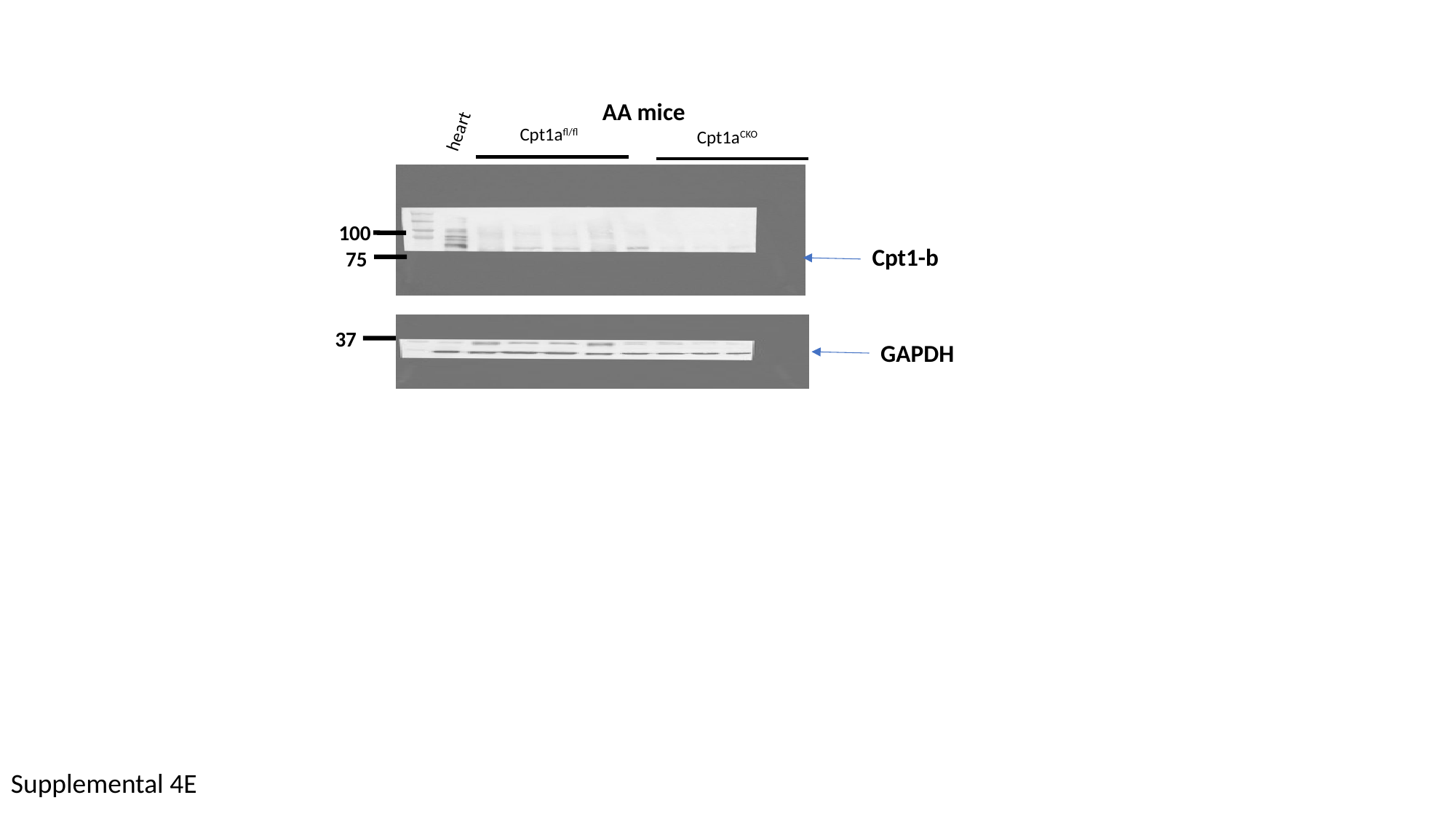

AA mice
heart
Cpt1afl/fl
Cpt1aCKO
100
Cpt1-b
75
37
GAPDH
Supplemental 4E

## Slide 10
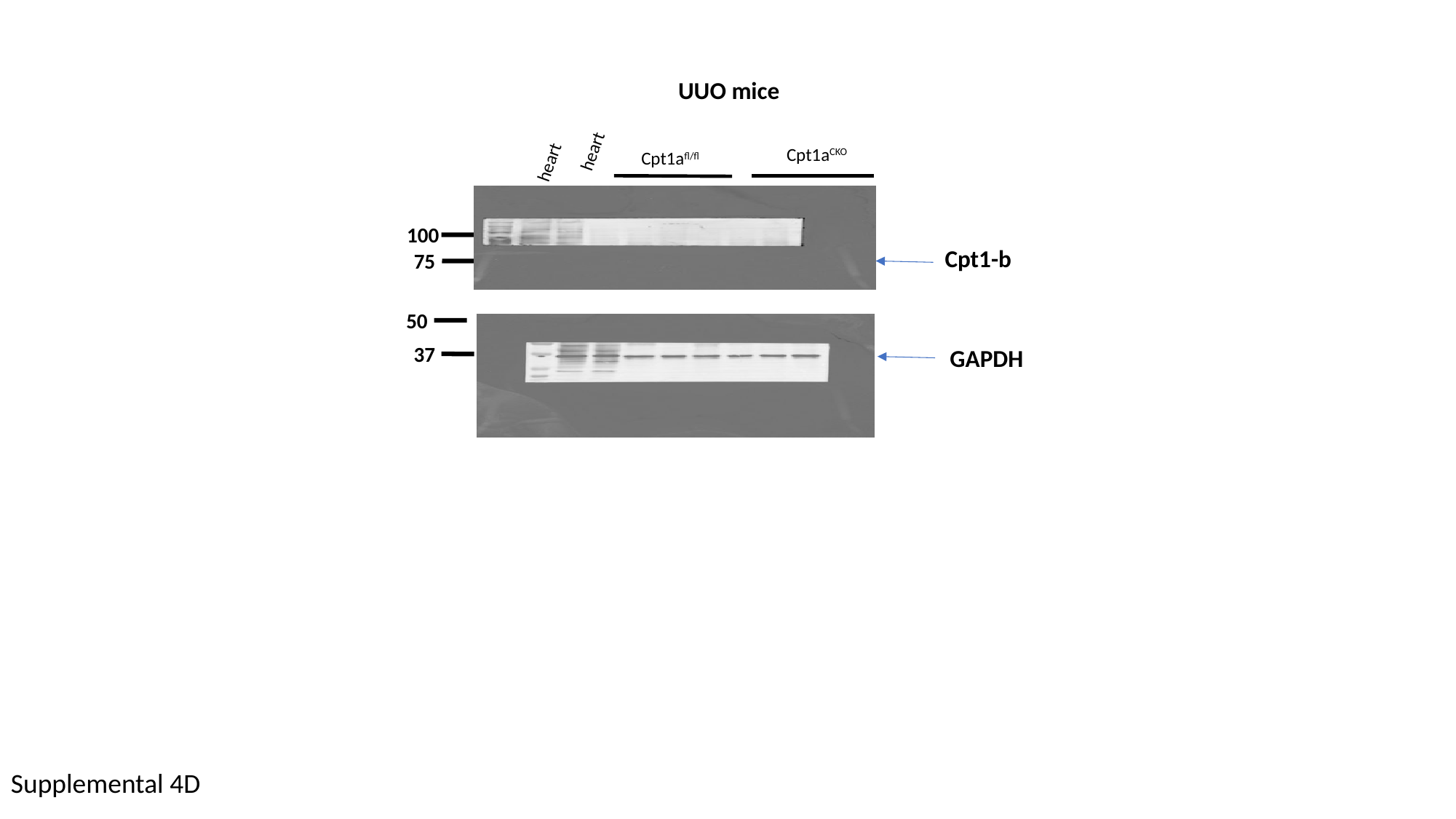

UUO mice
heart
Cpt1aCKO
Cpt1afl/fl
heart
100
Cpt1-b
75
50
37
GAPDH
Supplemental 4D

## Slide 11
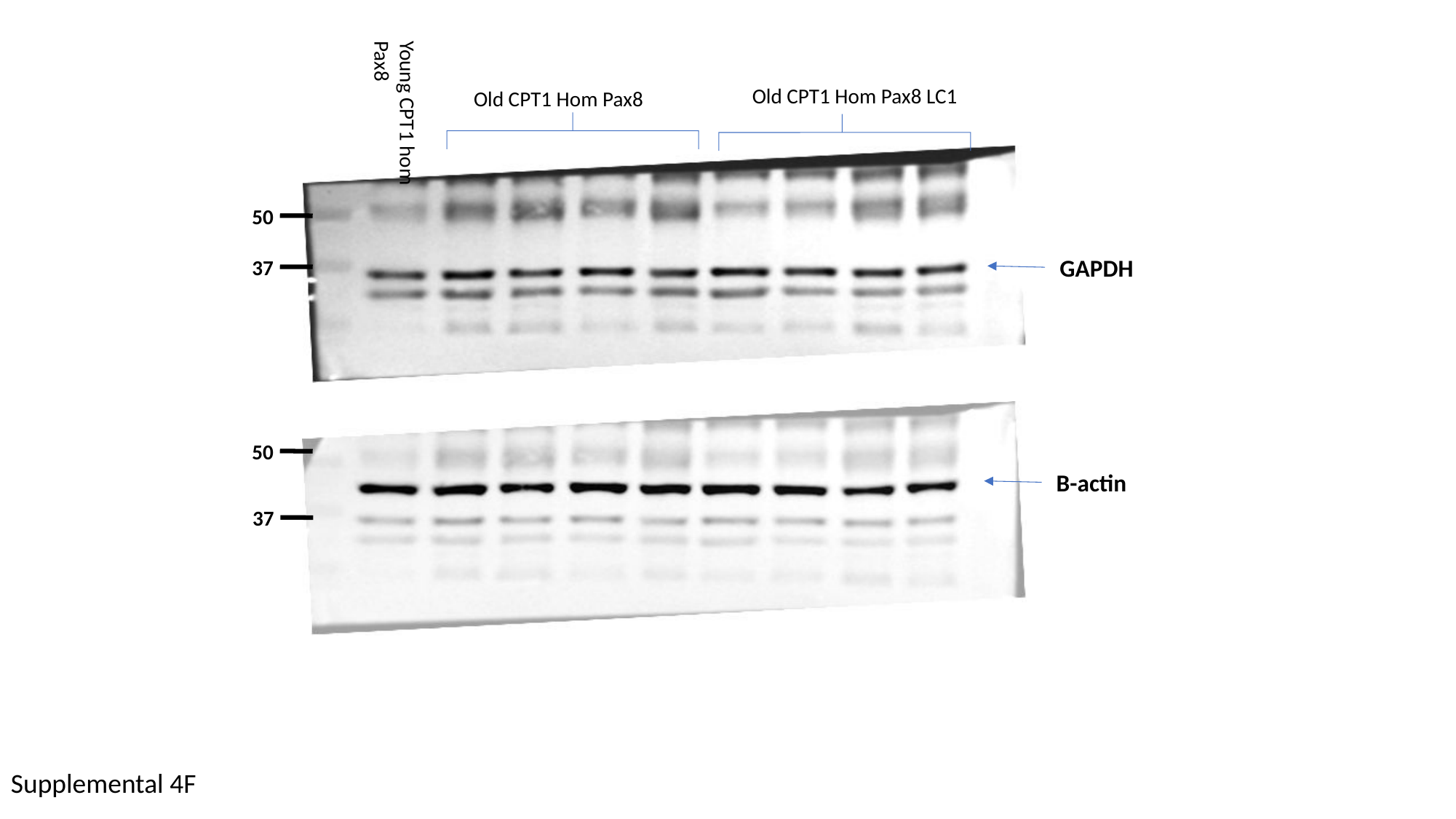

Young CPT1 hom Pax8
Old CPT1 Hom Pax8 LC1
Old CPT1 Hom Pax8
50
GAPDH
37
50
B-actin
37
Supplemental 4F
